# Integrating Clinical Signs at Presentation and Clinician's Non-analytical Reasoning in Prediction Models for Serious Bacterial Infection in Febrile Children Presenting to Emergency Department

**DOI:** 10.3389/fped.2022.786795

**Published:** 2022-04-25

**Authors:** Urzula Nora Urbane, Eva Petrosina, Dace Zavadska, Jana Pavare

**Affiliations:** ^1^Department of Pediatrics, Riga Stradins University, Riga, Latvia; ^2^Department of Pediatrics, Children's Clinical University Hospital, Riga, Latvia; ^3^Statistics Unit, Riga Stradins University, Riga, Latvia

**Keywords:** fever, serious bacterial infection, prediction model, non-analytical reasoning, gut feeling

## Abstract

**Objective:**

Development and validation of clinical prediction model (CPM) for serious bacterial infections (SBIs) in children presenting to the emergency department (ED) with febrile illness, based on clinical variables, clinician's “gut feeling,” and “sense of reassurance.

**Materials and Methods:**

Febrile children presenting to the ED of Children's Clinical University Hospital (CCUH) between April 1, 2017 and December 31, 2018 were enrolled in a prospective observational study. Data on clinical signs and symptoms at presentation, together with clinician's “gut feeling” of something wrong and “sense of reassurance” were collected as candidate variables for CPM. Variable selection for the CPM was performed using stepwise logistic regression (forward, backward, and bidirectional); Akaike information criterion was used to limit the number of parameters and simplify the model. Bootstrapping was applied for internal validation. For external validation, the model was tested in a separate dataset of patients presenting to six regional hospitals between January 1 and March 31, 2019.

**Results:**

The derivation cohort consisted of 517; 54% (*n* = 279) were boys, and the median age was 58 months. SBI was diagnosed in 26.7% (*n* = 138). Validation cohort included 188 patients; the median age was 28 months, and 26.6% (*n* = 50) developed SBI. Two CPMs were created, namely, CPM1 consisting of six clinical variables and CPM2 with four clinical variables plus “gut feeling” and “sense of reassurance.” The area under the curve (AUC) for receiver operating characteristics (ROC) curve of CPM1 was 0.744 (95% CI, 0.683–0.805) in the derivation cohort and 0.692 (95% CI, 0.604–0.780) in the validation cohort. AUC for CPM2 was 0.783 (0.727–0.839) and 0.752 (0.674–0.830) in derivation and validation cohorts, respectively. AUC of CPM2 in validation population was significantly higher than that of CPM1 [*p* = 0.037, 95% CI (−0.129; −0.004)]. A clinical evaluation score was derived from CPM2 to stratify patients in “low risk,” “gray area,” and “high risk” for SBI.

**Conclusion:**

Both CPMs had moderate ability to predict SBI and acceptable performance in the validation cohort. Adding variables “gut feeling” and “sense of reassurance” in CPM2 improved its ability to predict SBI. More validation studies are needed for the assessment of applicability to all febrile patients presenting to ED.

## Introduction

Fever in children remains one of the most common reasons for presentation to healthcare, accounting for up to 22% of visits to pediatric emergency departments (ED) (1–3). In most cases, the underlying cause of fever is mild or self-limiting infection, as the introduction of comprehensive vaccination programs covering pathogens such as *Haemophilus influenzae* type B and *Streptococcus pneumoniae* has provided a significant reduction in rates of bacteremia in young children (4, 5). However, in high-prevalence settings such as pediatric EDs, a significant proportion of febrile children [ranging from 4 to 25%, depending on the age group and setting (6–9)] will be diagnosed with a serious bacterial infection (SBI), defined by most studies as a range of infections including bacteremia, sepsis, pneumonia, bacterial meningitis, complicated urinary tract infection, acute osteomyelitis, septic arthritis, and others (7, 10–14), which may result in adverse outcomes or even death if not recognized early (15). Therefore, timely discrimination between children with a high or low risk for SBI at the ED setting is very challenging due to the multitude of patients. The high costs of investigation (16) and dissatisfaction in parents caused by long waiting times and painful procedures (17) increase the pressure on clinicians even further.

Several tools have been developed to aid clinicians in this complicated process. Clinical scoring systems, such as “Traffic light” assessment system by the National Institute for Health and Care Excellence (NICE) in the UK (18) and the Yale Observation Scale (YOS) (19), have been proposed. However, the value of YOS in predicting SBI has been very limited (20), and the “red” and “amber” clinical features in the NICE “Traffic light” system still failed to identify a significant proportion of children with serious infections in retrospective and prospective studies (21–23). A novel Liverpool Quick Sequential Organ Failure Assessment (LqSOFA) score adapted to pediatric patients has been developed and validated for the prediction of life-threatening infection and admission to intensive care unit showed a higher positive predictive value than the NICE “high-risk” features (24). However, assessment of an febrile child should not be limited to the recognition of critical conditions alone.

Furthermore, a number of clinical prediction models (CPMs) have been introduced to estimate the probability of SBI in febrile children (7, 8, 11–13, 21, 25–28), of which Feverkidstool, a model for prediction of pneumonia and other SBI, is the most extensively validated (11). CPMs including laboratory results in addition to clinical signs and symptoms (7, 11, 25, 27, 28) show far better performance in validation studies (29, 30) than CPMs with clinical variables alone (12, 13, 21). Despite these CPMs being more reliable, their application may be unfavorable in settings where rapid point-of-care tests (POCTs) are unavailable, and obtaining laboratory results requires extra time and effort from the personnel.

While it may be assumed that clinical signs, symptoms, and laboratory results should be the only evidence used by clinicians in their diagnostic reasoning (in accordance with the standards of evidence-based medicine), recent studies show wide recognition of the role of non-analytical, intuitive reasoning (31–34) and its added value in diagnosis of serious infections in children (12, 35), and gastrointestinal bleeding (36), cancer (37, 38), and other life- or limb-threatening conditions (39–41). Specifically, a clinician's “gut feeling of something being wrong” even if the clinical signs do not indicate serious illness has been associated with significantly increased risk for serious illness in febrile children presenting to primary care (35) and also identified as a key variable in a CPM derived from a primary care cohort (12). Another type of intuitive assessment, “sense of reassurance,” defined as feeling sure about the future course of the patient's illness, even when being unsure about the precise diagnosis (42), has been found useful in ruling out SBI in febrile children presenting to ED (43). Similarly, parental concern about an episode of febrile illness in their child being different than their previous illnesses has been associated with increased likelihood of SBI in primary care (12), although less so in children presenting to ED (43).

This study derived and validated two prediction models to aid pediatricians in recognition of SBI after initial assessment of febrile children presenting to ED, for guidance to more purposeful investigation and treatment. The value of non-analytical, intuitive components of diagnostic reasoning was evaluated by integrating objective clinical signs and symptoms with variables describing skilled intuition of a clinician (defined as “gut feeling” or “sense of reassurance”), and parental concern, in CPM applicable to ED patients, and comparing it to a model with objective variables alone.

## Materials and Methods

### Description of the Study Sites

This was a prospective observational study with single-center derivation of CPMs, after which a multi-center validation was performed. The derivation cohort was composed of febrile patients presenting to the Emergency Department of Children's Clinical University Hospital (CCUH) in Riga, Latvia, between April 1, 2017 and December 31, 2018. The validation cohort consisted of patients presenting to ED of one of six regional hospitals in Latvia, between January 1, 2019 and March 31, 2019.

CCUH is the only hospital in Latvia providing tertiary level of care exclusively for children. As a university hospital, it is the main national site of training for medical students and residents in general pediatrics and pediatric subspecialties. The patients presenting to ED of CCUH are children younger than 18 years, with problems related to childhood illness, trauma, foreign bodies, or other emergencies. The number of annual visits to ED is approximately sixty-five thousand, around nine thousand of which are febrile episodes. It has been estimated that approximately half of the febrile patients presenting to the ED of CCUH are non-urgent, and approximately 43.3% are discharged after initial examination, while nearly 30% remain at the ED for prolonged observation for up to 24 h, and close to 27% of febrile patients are hospitalized (44).

The validation cohort was enrolled at the emergency departments of six regional hospitals in Latvia: Liepājas Regionālā slimnı̄ca, Daugavpils Regionālā slimnı̄ca, Vidzemes Slimnı̄ca, Jēkabpils Regionālā slimnı̄ca, Ziemelkurzemes Regionālā slimnı̄ca, and Balvu un Gulbenes Slimnı̄icu apvienı̄ba. These hospitals provide secondary level of healthcare services for people of all age groups and have a pediatric department. The emergency departments of these hospitals are visited by children and adults alike, who present with various accidents and emergencies.

### Enrolment in the Derivation Cohort

Febrile patients presenting to the Emergency Department of Children's Clinical University Hospital (CCUH) in Riga, Latvia between April 1, 2017 and December 31, 2018 were enrolled in a prospective observational study. The majority of the derivation cohort were recruits to the European Union (EU) Horizon 2020 project “Personalised Risk Assessment in Febrile Illness to Optimise Real-Life Management Across the European Union” (PERFORM) (45). The main goal of the PERFORM project is to improve diagnosis and management of febrile patients, by identification and validation of promising new discriminators of bacterial and viral infection including transcriptomic and clinical phenotypic markers (45). This study also included an additional group of patients whose parents or guardians did not consent to participate in the PERFORM project for various reasons (mostly due to the required additional laboratory samples) but agreed to the collection and analysis of the clinical data and to participation in the parental questionnaire.

The inclusion and exclusion criteria were the same for both derivation and validation cohorts. The inclusion criteria were patients of any age older than 1 month but younger than 18 years with documented fever (axillary temperature ≥38°C) or history of fever, given that informed consent to participation in the study was obtained from the legal guardians and/or patients themselves (if aged 14 years and above).

Patients with chronic comorbidities that may increase risk for infection (such as diabetes mellitus, cystic fibrosis, presence of foreign body such as central venous catheter, organ transplant, severe malnutrition, etc.), patients with primary or secondary immunodeficiency, and patients transferred from other hospital with already established diagnosis and/or available investigation results were excluded from participation in the study.

Recruitment of patients required their parents to be approached by the research team; the days of enrolment were distributed evenly throughout the study period, including evenings, nights, weekends, and holidays, when all eligible patients were approached.

### Assessed Variables

Clinical signs and symptoms for potential inclusion in the clinical prediction model were selected from among those listed as alarming features in NICE “Traffic light” scoring system (18) and from those with identified strong association with SBI in febrile patients in a systematic literature review (20). In total, 27 clinical variables were assessed (the complete list of variables can be viewed in **Table 3** in *Results*). The body temperature at presentation was measured *via* axillary liquid-in-glass thermometer, in addition to recording parent-reported peak body temperature during the episode prior to presentation. Vital signs (heart rate, blood pressure, and oxygen saturation) were assessed by an electronic monitor, and respiratory rate was evaluated by the clinician during physical examination. Assessed heart rate and respiratory rate were evaluated according to age; the reference values for each variable are available in Supplementary Table 1 (46, 47). Poor peripheral circulation was defined as cold hands and feet and/or prolonged capillary refill time (48). Clinical impression of “ill/toxic appearance,” defined as child appearing pale, mottled, or cyanotic, lethargic or inconsolable, or showing signs of respiratory distress (tachypnoea, chest retractions, etc.) (49) was also noted. Clinical signs and symptoms were recorded in a standardized case report form, where the clinician noted the signs that were present; the signs and symptoms that were not noted were considered as absent by the research team.

In addition to noting clinical variables, the attending clinician was asked to fill a short questionnaire for assessment of “gut feeling,” defined as an intuitive feeling that the child may have a serious illness (“something is wrong”) (12, 35) and “sense of reassurance,” defined as a feeling that the child's illness is mild or self-limiting (42). The questionnaire was completed after physical examination of the child, before any additional investigation results were available. Both “gut feeling” and “sense of reassurance” were evaluated as “present,” “not sure,” or “absent” in case the clinician stated in the questionnaire that they did not experience “gut feeling.” In the statistical analyses coded as binary, “present” or “absent”/“not sure” was used.

The parents of enrolled patients were asked to fill a questionnaire evaluating their concern about the child during the particular episode of illness. Parental concern was defined as an impression that this episode of illness is different/more severe than the child's previous febrile episodes (12, 50) and was evaluated according to a 7-point Likert scale, where “definitely yes,” “most likely yes,” and “more likely yes than no” were interpreted as present, “difficult to say” was regarded as neutral, while “more likely no than yes,” “most likely no,” and “definitely no” were interpreted as absent. In statistical analysis, the evaluation “difficult to say” was coded equal to “absent.”

Both parental and clinicians' questionnaires were developed in collaboration with the Department of Public Health and Epidemiology of Riga Stradins University. The parental questionnaire was piloted in a small cohort of 26 patients, after which some alterations were made in questions unrelated to parental concern. The complete questionnaire can be viewed in the Supplementary File 1 (parental questionnaire in English) and Supplementary File 2 (parental questionnaire in Latvian). The contents of clinicians' questionnaire were discussed with experienced pediatricians, after which no changes were made. Introduction on completion of clinicians' questionnaire was provided to clinicians working at the ED of CCUH and the regional hospitals prior to the study. The complete clinicians' questionnaire can be viewed as Supplementary File 3 (English version) and Supplementary File 4 (Latvian version). As the questionnaires were considered as extensions of the main case report form for assessment of variables “gut feeling,” “sense of reassurance,” and “parental concern”; no validation procedures were performed.

### Outcome Definition

The primary outcome of interest was defined as presence or absence of SBI. SBI was defined as any of the infections displayed in Table 1 requiring hospitalization (for at least 24 h).

**Table 1:** Definitions and reference standards for SBI used in the study.

**No**.	**Type of infection**	**Reference standards**
1.	Bacteremia	A single bacterial pathogen identified in a blood culture
2.	Bacterial meningitis	Polymorphonuclear leukocytosis and bacterial pathogen identified in cerebrospinal fluid
3.	Pneumonia	An infiltrate on a chest X-ray identified by a pediatric radiologist
4	Urinary tract infection	Positive urine culture (10^5^ colony forming units (CFU) per ml of a single bacterial pathogen in a midstream urine sample or 10^4^ CFU/ml in a catheterized sample
5.	Bacterial soft tissue infections	Cellulitis/phlegmon/erysipelas/deep pus collection or abscess requiring hospitalization and systemic antibacterial therapy
6.	Bacterial gastroenteritis with dehydration	Bacterial pathogen identified in a stool sample of a patient with symptoms of acute gastroenteritis requiring hospitalization and intravenous rehydration
7.	Acute complicated appendicitis	Acute appendicitis with necrosis/perforation/peritonitis
8.	Acute osteomyelitis/septic arthritis	Pathogenic bacteria isolated from bone/joint aspirate OR osteomyelitis identified in MRI

The final diagnoses, either SBI or non-SBI, were made by the pediatricians directly involved in the care for the patient and extracted from medical records by the research team. All presumed SBIs were reviewed by the research team (U.N.U., D.Z., and J.P.), after the follow-up period. A diagnosis was classified as SBI if it met the criteria defined in Table 1.

Secondary outcomes were hospitalization, antibacterial treatment, and admission to pediatric Intensive Care Unit (ICU).

### Follow-Up

The patients were followed up until discharge from the hospital and further for up to 28 days from presenting to ED, to rule out or confirm development of SBI, initiation of antibiotics, or readmission to the hospital. For patients discharged from the hospital before day 28, the follow-up was arranged *via* telephone close to day 28 (on a working day, during working hours). Two call attempts were made by a member of the research team to contact the patient/guardians, after which no further attempts were made. If the research team failed to contact a patient, the possibility of readmission was ruled out by researching the patient on the hospital record system. In case the research team failed to contact a patient from the validation cohort (regional hospitals), information on possible admission to CCUH as the reference hospital was also ruled out. As the diagnosis of SBI for this study required hospitalization for at least 24 h due to one of infections meeting criteria for SBI, no patient without SBI was reclassified as SBI unless there was a readmission.

### Statistical Analysis

Variable selection for the clinical prediction model was performed using stepwise logistic regression (forward, backward, and bidirectional). A sample size of 500 subjects is recommended for derivation of CPMs *via* logistic regression of unknown number of variables for observational studies with large populations (51); another equation to estimate the sample size is 100 + 50*i*, where *i* refers to the number of independent variables selected for the final model. No data imputation for missing values was performed, and only cases with no missing data were used in logistic regression (complete case analysis). The aim of this study was to create a short, simple screening model; therefore, Akaike information criterion (AIC) was used to penalize for too many parameters.

Two clinical prediction models were created—one with clinical parameters (signs and symptoms) alone, and another, in which “gut feeling” and “sense of reassurance” were also included. For each of the two models, likelihood ratio (LR), Wald, and conditional selection criteria were used to assess the variety of regression models. The performance of the models was assessed by constructing a receiver operating characteristic (ROC) curve and calculation of sensitivity, specificity, positive and negative predictive values, and positive and negative likelihood ratios at different cut-off points in both derivation and validation cohorts. The statistical significance of the difference between the AUCs of the models was assessed by DeLong's test for two ROC curves.

Bootstrapping was used for assessment of the model's internal validity and correction for overoptimism. For external validation, the model was tested for the prediction of SBI in a separate dataset of patients presenting to one of six regional hospitals.

The statistical analysis was performed by using MS Excel and RStudio software version 1.4.1103.

## Results

### Demographics

In total, 517 patients presenting to the ED of CCUH were enrolled. Consent to participate in the PERFORM project was given by guardians of 385 patients, and additional 132 patients agreed to participate outside the PERFORM project. The proportion of male patients was 54% (*n* = 279). The age of the patients ranged from 1 month to 17 years and 11 months; the median age was 58 months. Forty-seven patients (9.1%) were younger than 1 year, and 261 children (50.5%) were younger than 5 years. Among patients with SBI, 58.0% (*n* = 80) were male, 49.3% (*n* = 68) were younger than 5 years, and 12.3% (*n* = 17) were younger than 12 months.

In regional hospitals, 188 patients were enrolled for validation of created CPMs. Boys composed 48.9% (*n* = 92) of the validation population. The median age of patients in the validation cohort was 28 months (range, 1 month to 16 years and 4 months). The proportion of patients younger than 12 months was 18.1% (*n* = 34), and 81.4% of patients (*n* = 153) were younger than 5 years. Only 36.0% (*n* = 18) of patients with SBI were male, 76.0% (*n* = 38) were younger than 5 years, and 22.0% (*n* = 11) were younger than 12 months.

### Follow-Up

In derivation cohort, 300 patients (58.0%) were contacted *via* telephone. Eight patients (1.5%) were still at hospital on/close to day 28 since admission. Development of SBI in the remaining 209 patients (40.4%) was ruled out by researching data on hospital readmission in CCUH database.

In validation cohort, 125 patients (66.5%) were contacted *via* telephone, and hospital readmission on 63 patients (33.5%) was ruled out by researching their data in the databases of the regional hospital of admission and CCUH as the potential reference hospital.

### Outcomes

Of all children included in the research cohort, 26.7% (*n* = 138) were diagnosed with SBI. The final diagnoses of the patients are summarized in Table 2. All patients with SBI were hospitalized for at least 24 h and received antibiotics; 31 of these patients (22.5%) were hospitalized in the ICU. The duration of hospitalization in patients with SBI ranged from 1 to 44 days (median, 5 days).

**Table 2:** Final diagnoses in derivation cohort (CCUH) and validation cohort (regional hospitals).

**Diagnosis**	**CCUH** ***n*** **(%)**	**Regional hospitals** ***n*** **(%)**
SBI present	138 (26.7%)	50 (26.6%)
Pneumonia	68 (13.2%)	34 (18.1%)
Urinary tract infection	22 (4.3%)	14 (7.4%)
Acute complicated appendicitis, peritonitis	9 (1.7%)	0 (0%)
Frontitis, orbital cellulitis, mastoiditis	3 (0.6%)	0 (0%)
Invasive soft tissue infection (phlegmon, cellulitis, abscess)	8 (1.5%)	0 (0%)
Acute osteomyelitis/septic arthritis	10 (1.9%)	0 (0%)
Bacterial gastroenteritis	7 (1.4%)	2 (1.1%)
Bacterial meningitis (incl. meningococcal)	4 (0.8%)	0 (0%)
Meningococcal bacteremia without meningitis	2 (0.4%)	0 (0%)
Bacteremia with shock or multiorgan injury	2 (0.4%)	0 (0%)
Other bacteremia	3 (0.6%)	0 (0%)
SBI absent	379 (73.3%)	138 (73.4%)
Upper respiratory tract infections (incl. nasopharyngitis, conjunctivitis, stomatitis, gingivitis, non-specific)	69 (13.3%)	29 (15.4%)
Tonsillitis/pharyngitis	75 (14.5%)	25 (13.3%)
Acute laryngitis (croup)	2 (0.4%)	4 (2.1%)
Acute otitis media	9 (1.7%)	5 (2.7%)
Parotitis	3 (0.6%)	0 (0%)
Infectious mononucleosis	7 (1.4%)	2 (1.1%)
Influenza	29 (5.6%)	24 (12.8%)
Lower respiratory tract infection (bronchitis/bronchiolitis)	37 (7.2%)	36 (19.1%)
Scarlet fever	5 (1.0%)	1 (0.5%)
Acute gastroenteritis	41 (7.9%)	6 (3.2%)
Acute uncomplicated appendicitis	8 (1.5%)	0 (0%)
Aseptic meningitis, encephalitis	11 (2.1%)	0 (0%)
Viral syndrome	27 (5.2%)	3 (1.6%)
Unspecified uncomplicated bacterial infection	33 (6.4%)	2 (1.1%)
Inflammatory/autoimmune	4 (0.8%)	1 (0.5%)
Unspecified diagnosis	10 (1.9%)	0 (0%)
Other	9 (1.7%)	0 (0%)

Of the 379 patients who did not develop SBI, 191 (50.4%) received or were prescribed antibiotics, 228 (60.2%) were hospitalized, and five patients (1.3%) were hospitalized in ICU. The median duration of hospitalization among patients without SBI was 2 days, ranging from 0 to 25 days.

In the validation population consisting of 188 patients from regional hospitals, 26.6% of patients (*n* = 50) developed SBI. Data on demographics, assessed variables, and outcomes can be viewed in detail in Supplementary File 5 (MS Excel database).

### Predictor Variables

In the derivation population (CCUH patients), data on 30 variables were collected, which are listed in Table 3. In 70 cases, data on the highest temperature within the episode were missing; in 7 cases, the duration of fever was unknown, and in 4 cases, the heart rate was not noted.

**Table 3:** Frequency of predictor variables in derivation population.

**Variable**	**Present,** ***n*** **(%)**	**Present in SBI,** ***n*** **(%)**	**Present in non-SBI,** ***n*** **(%)**	**Missing**
T ≥ 40°C (reported by parents)	115 (22.2%)	37 (26.8%)	78 (20.6%)	70
Fever ≥ 3 days	206 (39.8%)	68 (49.3%)	138 (36.4%)	7
Tachycardia	135 (26.1%)	38 (27.5%)	97 (25.6%)	4
Ill/toxic appearance	140 (27.1%)	68 (49.3%)	72 (19.0%)	0
Drowsiness	138 (26.7%)	49 (35.5%)	89 (23.5%)	0
Lethargy	21 (4.1%)	11 (8.0%)	10 (2.6%)	0
Irritability	43 (8.3%)	11 (8.0%)	32 (8.4%)	0
Grunting	21 (4.1%)	10 (7.2%)	11 (2.9%)	0
Inconsolable crying	20 (3.9%)	7 (5.1%)	13 (3.4%)	0
Reduced appetite	258 (49.9%)	71 (51.4%)	187 (49.3%)	0
Refusal of food	101 (19.5%)	29 (21.0%)	72 (19.0%)	0
Refusal to drink	115 (22.2%)	23 (16.7%)	92 (24.3%)	0
Reduced urine output	98 (19.0%)	30 (21.7%)	68 (17.9%)	0
Reduced skin turgor	63 (12.2%)	19 (13.8%)	44 (11.6%)	0
Cyanosis	0 (0.0%)	0 (0.0%)	0 (0.0%)	0
Tachypnoea	78 (15.1%)	38 (27.5%)	40 (10.6%)	0
Abnormal breath sounds	76 (14.7%)	35 (25.4%)	41 (10.8%)	0
Reduced breath sounds	28 (5.4%)	17 (12.3%)	11 (2.9%)	0
Shortness of breath	22 (4.3%)	10 (7.2%)	12 (3.2%)	0
Chest retractions	25 (4.8%)	15 (10.9%)	10 (2.6%)	0
Poor peripheral circulation	32 (6.2%)	20 (14.5%)	12 (3.2%)	0
Meningeal signs	15 (2.9%)	4 (2.9%)	11 (2.9%)	0
Non-blanching rash	24 (4.6%)	8 (5.8%)	16 (4.2%)	0
Seizures	7 (1.4%)	2 (1.4%)	5 (1.3%)	0
Hypotension	6 (1.2%)	4 (2.9%)	2 (0.5%)	0
Loss of consciousness	4 (0.8%)	2 (1.4%)	2 (0.5%)	0
Hypothermia	1 (0.2%)	1 (0.7%)	0 (0.0%)	0
“Gut feeling” of something wrong	104 (20.1%)	46 (33.3%)	58 (15.3%)	161
Sense of reassurance	102 (19.7%)	5 (3.6%)	97 (25.6%)	162
Parental concern	171 (33.1%)	47 (34.1%)	124 (32.7%)	250

In 161 and 162 cases, respectively, data on clinician's “gut feeling” and “sense of reassurance” were missing due to inability of the doctor to complete the clinician's questionnaire within the specified time frame (before investigation results became available). Nearly half of the parents (250) failed to submit parental questionnaire to the research team. Despite mild association of parental concern with SBI [OR (95% CI) = 1.90 (1.01–3.57), *p* = 0.046], due to large amount of missing data, inclusion of this variable was decided against, as analysis of remaining data also showed poor diagnostic value [OR (+) (95% CI) = 1.22 (1.02–1.47)].

Highest reported temperature equal to or above 40°C was also not associated with SBI in our study population [OR (95% CI) =1.55 (0.97–2.46), *p* > 0.05]. As this variable was missing in 70 cases, it was also excluded. Prior to exclusion, the relevance of body temperature as a predictor variable was ruled out by entering several thresholds (above 39.0°C, above 39.5°C, and above 40.0°C) separately in logistic regression analysis. In none of the cases, the body temperature was selected as a variable, nor did it change the other selected variables. Variables “cyanosis,” “hypotension,” “loss of consciousness,” and “hypothermia” were further excluded, as they were present in 1% of population or less. The remaining variables were considered for derivation of the model.

Two CPMs were created. In derivation of the first model (CPM 1), the variables “gut feeling” and “sense of reassurance” were not entered, and the model was based on clinical signs and symptoms alone. The second model (CPM 2) included these variables. Due to missing data, derivation of CPM 1 was possible from 511 complete cases of the CCUH patients (26.4% of whom had SBI), while CPM 2 was based on 345 complete cases (with 23.1% prevalence of SBI) in whom all the necessary variables were noted. For easier comparability of the models, both CPM1 and CPM2 were derived from the cohort of 345 patients without missing variables.

Assessment of variety of possible models in each case by likelihood ratio, Wald, and conditional selection criteria yielded similar results and did not provide significant improvement. Correction for optimism was performed by applying the model to 100,000 bootstrap samples of the data. The variables selected for the best model according to AIC criteria for CPM 1 are reflected in Table 4.

**Table 4:** Variables of clinical prediction model 1.

**Variables**	**Regression coefficient**	**Standard error**	**Odds ratio (95% CI)**
Ill/toxic appearance	0.84	0.33	2.31 (1.25–4.66)
Refusal to drink	−0.58	0.37	0.56 (0.27–1.17)
Tachypnoea	0.73	0.39	2.07 (0.98–4.64)
Abnormal breath sounds	0.62	0.43	1.86 (0.84–4.56)
Reduced breath sounds	1.56	1.02	4.74 (1.34–18.84)
Poor peripheral circulation	1.31	0.93	3.69 (0.89–17.77)

In CPM 1, ill/toxic appearance, tachypnoea, abnormal breath sounds, reduced breath sounds, and poor peripheral circulation increased the likelihood of SBI, while refusal to drink decreased the odds to develop SBI.

Table 5 reflects the variables selected according to AIC criteria as best for CPM 2. In CPM 2, tachypnoea, reduced breath sounds, poor peripheral circulation, and “gut feeling” increased the odds for SBI, while refusal to drink and “sense of reassurance” lowered the odds for being diagnosed with SBI.

**Table 5:** Variables of clinical prediction model 2.

**Characteristics**	**Regression coefficient**	**Standard error**	**Odds ratio (95% CI)**
Refusal to drink	−0.51	0.36	0.60 (0.30–1.24)
Tachypnoea	0.85	0.39	2.34 (1.14–5.19)
Reduced breath sounds	1.48	1.00	4.37 (1.27–15.91)
Poor peripheral circulation	0.96	0.85	2.61 (0.65–11.02)
“Gut feeling”	0.64	0.32	1.90 (1.04–3.68)
“Sense of reassurance”	−1.63	1.41	0.20 (0.06–0.66)

### Performance of Clinical Prediction Models

**Figure 1:**
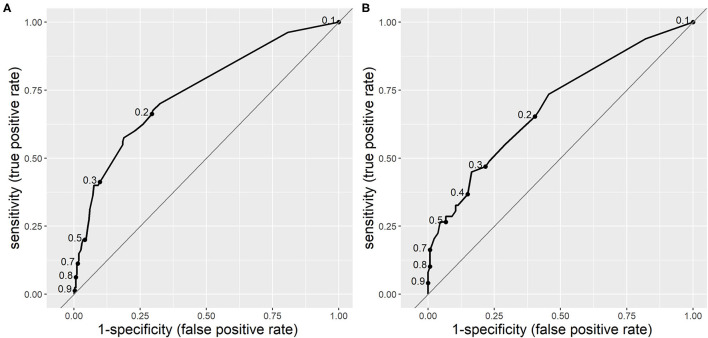
Receiver operating characteristic curves of clinical prediction model 1 (CPM 1) for risk of serious bacterial infections (SBIs) in derivation **(A)** and validation **(B)** populations. The dots on the curves represent sensitivity and specificity at different cut-off points.

The area under the curve (AUC) for the receiver operating characteristic (ROC) curve of CPM 1 was 0.744 (95% CI 0.683–0.805), which is considered as moderate. In the validation population, the AUC for CPM 1 was 0.692 (95% CI 0.604–0.780), which is an acceptable difference. The ROC curves of CPM 1 in both derivation and validation populations are shown in Figure 1.

**Figure 2:**
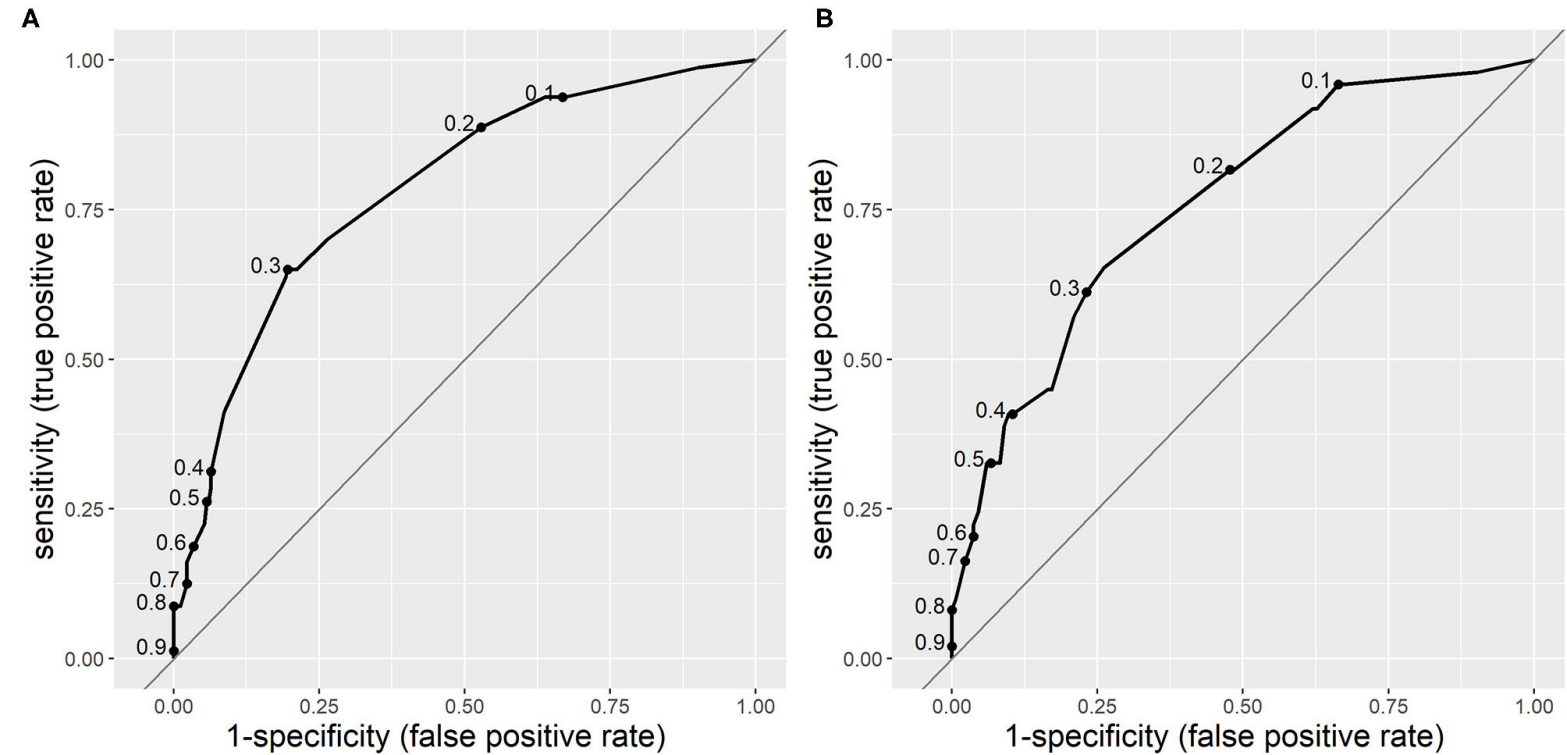
Receiver operating characteristic curves of clinical prediction model 2 (CPM 2) for risk of serious bacterial infections (SBIs) in derivation **(A)** and validation **(B)** populations. The dots on the curves represent sensitivity and specificity at different cut-off points.

The ROC area under the curve for CPM 2 was 0.783 (95% CI 0.727–0.839), which is also moderate, but surpasses that of CPM 1. In the validation population, the AUC was slightly lower than in the research population−0.752 (95% CI 0.674–0.830), which is also an acceptable difference. Figure 2 displays the ROC curves of CPM 2 in derivation and validation populations.

According to DeLong's test for two ROC curves, the improvement of AUC of CPM2 in the validation population over that of CPM1 was statistically significant [*p* = 0.037, 95%CI (−0.129; −0.004)].

**Figure 3:**
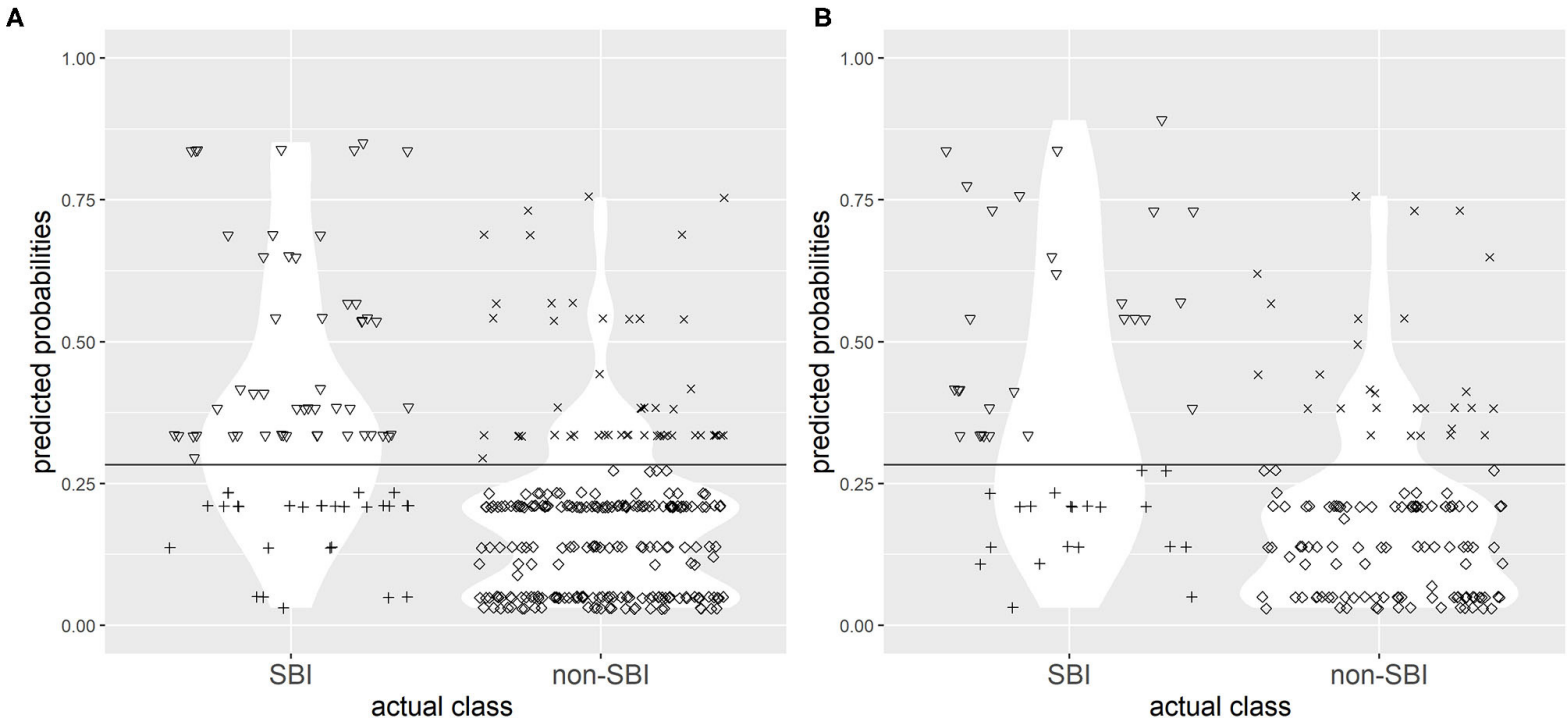
Confusion matrix for discrimination between subjects with SBI and without SBI by clinical prediction model 1 (CPM 1) in research **(A)** and validation **(B)** populations with the chosen cut-off value of 0.219. Symbols: ▽ true positives; + false negatives; x false positives; ♢ true negatives. The horizontal line represents the cut-off value.

**Figure 4:**
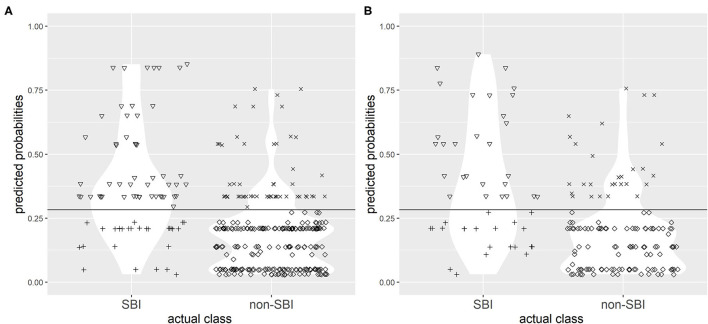
Confusion matrix for discrimination between subjects with SBI and without SBI by clinical prediction model 2 (CPM 2) in research **(A)** and validation **(B)** populations with the chosen cut-off value of 0.283. Symbols: ▽ true positives; + false negatives; x false positives; ♢ true negatives. The horizontal line represents the cut-off value.

The choice of single best cut-off point values proved to be problematic for both CPMs. A cut-off point value of 0.268 to discriminate between the two groups (SBI and non-SBI) was set for CPM 1 based on Youden's index to provide highest possible sensitivity and specificity, and a cut-off value of 0.283 was set for CPM 2. Figures 3, 4 illustrate the results of application of CPM1 and CPM2, respectively, to both derivation and validation cohorts, showing the distribution of patients with and without SBI around the estimated cut-off line.

It was evident that choice of a single cut-off point, even with best possible sensitivity and specificity, resulted in a high concentration of patients near the cut-off points who were falsely predicted as either SBI or non-SBI.

The sensitivity of CPM 1 in the research cohort at this chosen cut-off level was 57.5% (95% CI 45.9–68.5%), the specificity was 81.% (95% CI 75.9–85.7%), and the accuracy of the model was 75.7%. The model missed 34 (42.5%) cases with SBI, which were instead predicted as non-SBI. In the validation cohort, the model (at the chosen cut-off level) had 49.0% sensitivity (95% CI 34.4%−63.7%), 76.9% specificity (95% CI 68.8%−83.7%), and 69.4% accuracy. However, 25 (51.0%) patients with SBI were falsely predicted as non-SBI by the model.

Likewise, application of the chosen cut-off level to CPM 2 yielded a sensitivity of 65.0% (95% CI 53.5–75.3%), specificity of 80.4% (95% CI 75.0–85.0%), and accuracy of 76.8% in research population. Twenty-eight (35.0%) of the cases with SBI were falsely identified as non-SBI. In the validation population, use of the cut-off resulted in a sensitivity of 56.2% (95% CI 41.2–70.5%), 79% specificity (95% CI 71.0–85.5%), and 72.9% accuracy, although 21 (43.8%) of patients with SBI were falsely identified as non-SBI.

The performance of CPM 1 (sensitivity, specificity, positive and negative predictive values, and positive and negative likelihood ratios) in derivation and validation populations are shown in Table 6, while the performance of CPM 2 is reflected in Table 7.

**Table 6:** Diagnostic performance of CPM 1 at different cut-off points in derivation and validation cohorts.

**Cut-off**	**Sensitivity** **(95% CI)**	**Specificity** **(95% CI)**	**PPV** **(95% CI)**	**NPV** **(95% CI)**	**LR (+)** **(95% CI)**	**LR (–)** **(95% CI)**
**Derivation cohort (CCUH)**						
0.10	0.96 (0.89–0.99)	0.19 (0.15–0.25)	0.26 (0.21–0.32)	0.94 (0.85–0.99)	1.19 (1.11–1.28)	0.19 (0.06–0.61)
0.20	0.62 (0.51–0.73)	0.74 (0.68–0.79)	0.42 (0.33–0.51)	0.87 (0.82–0.91)	2.40 (1.84–3.13)	0.51 (0.38–0.68)
0.30	0.40 (0.29–0.52)	0.91 (0.87–0.94)	0.57 (0.43–0.70)	0.83 (0.79–0.87)	4.42 (2.77–7.04)	0.66 (0.55–0.79)
0.40	0.36 (0.26–0.48)	0.93 (0.89–0.96)	0.60 (0.45, 0.74)	0.83 (0.78–0.87)	5.06 (3.00–8.52)	0.69 (0.58–0.81)
0.50	0.20 (0.12–0.30)	0.96 (0.93–0.98)	0.62 (0.41–0.80)	0.80 (0.75–0.84)	5.30 (2.50–11.21)	0.83 (0.74–0.93)
0.60	0.20 (0.12–0.30)	0.97 (0.94–0.98)	0.64 (0.43–0.82)	0.80 (0.75–0.84)	5.89 (2.71–12.81)	0.83 (0.74–0.93)
0.70	0.11 (0.05–0.20)	0.98 (0.96–1.00)	0.69 (0.39–0.91)	0.79 (0.74–0.83)	7.45 (2.36–23.56)	0.90 (0.83–0.98)
**Validation cohort (regional hospitals)**						
0.10	0.94 (0.83–0.99)	0.18 (0.12–0.25)	0.29 (0.22–0.37)	0.89 (0.71–0.98)	1.14 (1.03–1.27)	0.34 (0.11–1.08)
0.20	0.61 (0.46–0.75)	0.64 (0.55–0.72)	0.38 (0.28–0.50)	0.82 (0.73–0.89)	1.71 (1.24–2.35)	0.60 (0.42–0.88)
0.30	0.45 (0.31–0.60)	0.84 (0.76–0.89)	0.50 (0.35–0.65)	0.81 (0.73–0.87)	2.73 (1.67–4.47)	0.66 (0.51–0.86)
0.40	0.37 (0.23–0.52)	0.85 (0.78–0.91)	0.47 (0.31–0.64)	0.79 (0.71–0.85)	2.46 (1.43–4.25)	0.74 (0.59–0.93)
0.50	0.27 (0.15–0.41)	0.94 (0.89–0.97)	0.62 (0.38–0.82)	0.78 (0.71–0.84)	4.44 (1.96–10.07)	0.78 (0.66–0.93)
0.60	0.27 (0.15–0.41)	0.94 (0.89–0.97)	0.62 (0.38–0.82)	0.78 (0.71–0.84)	4.44 (1.96–10.07)	0.78 (0.66–0.93)
0.70	0.16 (0.07–0.30)	0.99 (0.96–1.00)	0.89 (0.52–1.00)	0.76 (0.69–0.83)	21.88 (2.81–170.44)	0.84 (0.74–0.95)

**Table 7:** Diagnostic performance of CPM 2 at different cut-off points in derivation and validation cohorts.

**Cut-off**	**Sensitivity** **(95% CI)**	**Specificity** **(95% CI)**	**PPV** **(95% CI)**	**NPV** **(95% CI)**	**LR (+)** **(95% CI)**	**LR (–)** **(95% CI)**
**Derivation cohort (CCUH)**						
0.10	0.94 (0.86–0.98)	0.33 (0.28–0.39)	0.30 (0.24–0.36)	0.95 (0.88–0.98)	1.40 (1.27–1.55)	0.19 (0.08–0.45)
0.20	0.89 (0.80–0.95)	0.47 (0.41–0.53)	0.34 (0.27–0.40)	0.93 (0.88–0.97)	1.68 (1.46–1.93)	0.24 (0.13–0.45)
0.30	0.64 (0.52–0.74)	0.81 (0.75–0.85)	0.50 (0.40–0.60)	0.88 (0.83–0.92)	3.31 (2.46–4.46)	0.45 (0.33–0.60)
0.40	0.31 (0.21–0.43)	0.94 (0.90–0.96)	0.60 (0.43–0.74)	0.82 (0.77–0.86)	4.87 (2.77–8.55)	0.73 (0.63–0.85)
0.50	0.26 (0.17–0.37)	0.94 (0.91–0.97)	0.58 (0.41–0.74)	0.81 (0.76–0.85)	4.64 (2.51–8.57)	0.78 (0.68–0.89)
0.60	0.16 (0.09–0.26)	0.98 (0.95–0.99)	0.68 (0.43–0.87)	0.79 (0.75–0.84)	7.18 (2.82–18.27)	0.86 (0.78–0.95)
0.70	0.09 (0.04–0.17)	0.99 (0.97–1.00)	0.70 (0.35–0.93)	0.78 (0.73–0.83)	7.73 (2.05–29.20)	0.92 (0.86–0.99)
**Validation cohort (regional hospitals)**						
0.10	0.96 (0.86–1.00)	0.34 (0.26–0.42)	0.35 (0.27–0.43)	0.96 (0.85–0.99)	1.44 (1.26–1.65)	0.12 (0.03–0.48)
0.20	0.82 (0.68–0.91)	0.52 (0.43–0.61)	0.38 (0.29–0.49)	0.89 (0.79–0.95)	1.71 (1.37–2.13)	0.35 (0.19–0.65)
0.30	0.57 (0.42–0.71)	0.79 (0.71–0.86)	0.50 (0.36–0.64)	0.83 (0.76–0.89)	2.73 (1.82–4.12)	0.54 (0.39–0.76)
0.40	0.41 (0.27–0.56)	0.90 (0.83–0.94)	0.59 (0.41–0.75)	0.81 (0.73–0.87)	3.91 (2.15–7.11)	0.66 (0.52–0.84)
0.50	0.33 (0.20–0.48)	0.94 (0.89–0.97)	0.67 (0.45–0.84)	0.79 (0.72–0.85)	5.47 (2.50–11.97)	0.72 (0.59–0.87)
0.60	0.20 (0.10–0.34)	0.96 (0.92–0.99)	0.67 (0.38–0.88)	0.77 (0.70–0.83)	5.47 (1.97–15.20)	0.83 (0.71–0.96)
0.70	0.16 (0.07–0.30)	0.98 (0.94–1.00)	0.73 (0.39–0.94)	0.76 (0.69–0.82)	7.29 (2.02–26.38)	0.86 (0.75–0.97)

There was a significant gap between the risk thresholds with an optimal rule-in and rule-out values for SBI. For CPM 1, a 10% risk threshold had a sensitivity of 96% (95% CI 89–99%) and negative likelihood ratio of 0.19 (95%CI 0.06–0.61) in derivation population, while the positive likelihood ratio was low. By contrast, a cut-off of 0.4 was sufficient for ruling-in SBI [LR (+) (95% CI) = 5.06 (3.00–8.52), specificity (95% CI) = 93% (89–96%)], although with a low sensitivity of 36% (95% CI 26–48%). In the validation population, the sensitivity at the low-risk threshold of 10% was 94% (95% CI 83–99%), while the specificity of the 40% threshold was lower than in the derivation population (85%, 95% CI 78–91%).

Similar gap was evident for CPM 2, in which the recommended cut-off for ruling out SBI was 0.1, while a cut-off of 0.6 was optimal for ruling-in SBI, which yielded similar sensitivities and specificities in both cohorts.

To simplify the clinical applicability of the derived CPMs, we chose CPM 2 as the superior model according to its AUC in both derivation and validation populations, and a clinical score was created. The number of points in the score attributed to each variable was proportional to the regression coefficient, meaning that variables with negative regression coefficients were given negative points. To avoid negative total result, four points were added to the total sum of points, thus creating a range of 0–12 possible points. The variables and their attributed points in the score are reflected in Table 8.

**Table 8:** Clinical score to assess the risk for serious bacterial infection.

**Variables**	**Regression coefficient**	**Points if present**	**Points if absent**
Refusal to drink	−0.51	−1	0
Tachypnoea	0.85	2	0
Reduced breath sounds	1.48	3	0
Poor peripheral circulation	0.96	2	0
“Gut feeling”	0.64	1	0
“Sense of reassurance”	−1.63	−3	0
Total		Sum of points +4*	

* *Four points are added to the total sum of points to avoid negative result*.

The scoring system was subsequently applied to the derivation population and its performance assessed in the validation cohort. The sensitivities, specificities, positive and negative predictive values, and positive and negative likelihoods at different score cut-off values are reflected in Table 9.

**Table 9:** Diagnostic performance of scoring system based on CPM 2 at different cut-off score values in derivation and validation cohorts.

**Cut-off**	**Sensitivity** **(95% CI)**	**Specificity** **(95% CI)**	**PPV** **(95% CI)**	**NPV** **(95% CI)**	**LR (+)** **(95% CI)**	**LR (–)** **(95% CI)**
**Derivation cohort (CCUH)**						
≥1 point	0.99 (0.93–1.00)	0.10 (0.065–0.14)	0.28 (0.24–0.26)	0.96 (0.78–1.00)	1.09 (1.04–1.15)	0.13 (0.02–0.92)
≥2 points	0.94 (0.86–0.98)	0.33 (0.27–0.39)	0.30 (0.28–0.32)	0.95 (0.88–0.98)	1.40 (1.26–1.54)	0.19 (0.08–0.45)
≥3 points	0.94 (0.86–0.98)	0.33 (0.28–0.39)	0.30 (0.28–0.32)	0.95 (0.88–0.98)	1.40 (1.27–1.55)	0.19 (0.08–0.45)
≥4 points	0.89 (0.80–0.95)	0.47 (0.41–0.53)	0.34 (0.31–0.37)	0.93 (0.88–0.96)	1.68 (1.46–1.93)	0.24 (0.13–0.45)
≥5 points	0.65 (0.54–0.75)	0.79 (0.73–0.84)	0.48 (0.41–0.55)	0.88 (0.85–0.91)	3.08 (2.32–4.08)	0.44 (0.33–0.60)
≥6 points	0.41 (0.30–0.53)	0.91 (0.87–0.94)	0.60 (0.47–0.70)	0.84 (0.81–0.86)	4.75 (2.97–7.60)	0.64 (0.53–0.78)
≥7 points	0.26 (0.17–0.37)	0.94 (0.91–0.97)	0.58 (0.43–0.72)	0.81 (0.79–0.83)	4.64 (2.51–8.57)	0.78 (0.68–0.89)
≥8 points	0.16 (0.09–0.26)	0.98 (0.95–0.99)	0.68 (0.46–0.85)	0.80 (0.79–0.81)	7.18 (2.82–18.27)	0.86 (0.78–0.95)
≥9 points	0.09 (0.04–0.17)	0.99 (0.97–1.00)	0.70 (0.38–0.90)	0.78 (0.77–0.79)	7.73 (2.05–29.20)	0.92 (0.86–0.99)
**Validation cohort (Regional hospitals)**						
≥1 point	0.98 (0.89–1.00)	0.10 (0.05–0.16)	0.28 (0.27–0.30)	0.93 (0.64–0.99)	1.08 (1.01–1.16)	0.21 (0.03–1.57)
≥2 points	0.96 (0.86–1.00)	0.33 (0.25–0.41)	0.34 (0.31–0.37)	0.96 (0.85–0.99)	1.43 (1.25–1.63)	0.12 (0.03–0.49)
≥3 points	0.96 (0.86–1.00)	0.34 (0.26–0.42)	0.35 (0.32–0.38)	0.98 (0.85–0.99)	1.44 (1.26–1.65)	0.12 (0.03–0.48)
≥4 points	0.82 (0.68–0.91)	0.51 (0.43–0.60)	0.38 (0.33–0.43)	0.89 (0.81–0.93)	1.68 (1.35–2.10)	0.36 (0.19–0.66)
≥5 points	0.61 (0.46–0.75)	0.77 (0.69–0.84)	0.49 (0.40–0.59)	0.84 (0.79–0.89)	2.65 (1.81–3.87)	0.50 (0.35–0.73)
≥6 points	0.45 (0.31–0.60)	0.83 (0.75–0.89)	0.49 (0.37–0.61)	0.80 (0.76–0.84)	2.62 (1.61–4.25)	0.67 (0.51–0.87)
≥7 points	0.33 (0.20–0.48)	0.93 (0.88–0.97)	0.64 (0.46–0.79)	0.79 (0.76–0.82)	4.86 (2.30–10.27)	0.72 (0.59–0.88)
≥8 points	0.20 (0.10–0.34)	0.96 (0.92–0.99)	0.67 (0.42–0.85)	0.77 (0.74–0.79)	5.47 (1.97–15.20)	0.83 (0.71–0.96)
≥9 points	0.16 (0.07–0.30)	0.98 (0.94–1.00)	0.73 (0.42–0.91)	0.76 (0.74–0.78)	7.29 (2.02–26.39)	0.86 (0.75–0.97)

Basing on the sensitivity, specificity, and positive and negative likelihood ratio in derivation cohort, patients with score value of 3 points or less were stratified in a low-risk category for SBI, while patients who were assessed as reaching 6 or more points—into high-risk category. Patients with 4 or 5 points were classified as belonging to the “gray area.” This interpretation of the score had adequate performance in the validation population as well, with equal rule-out values for low-risk categories, while rule-in threshold for high-risk category in validation population was higher than in derivation cohort. Considering the goal for the model of reducing the number of missed cases of SBI, this was viewed as optimal.

**Figure 5:**
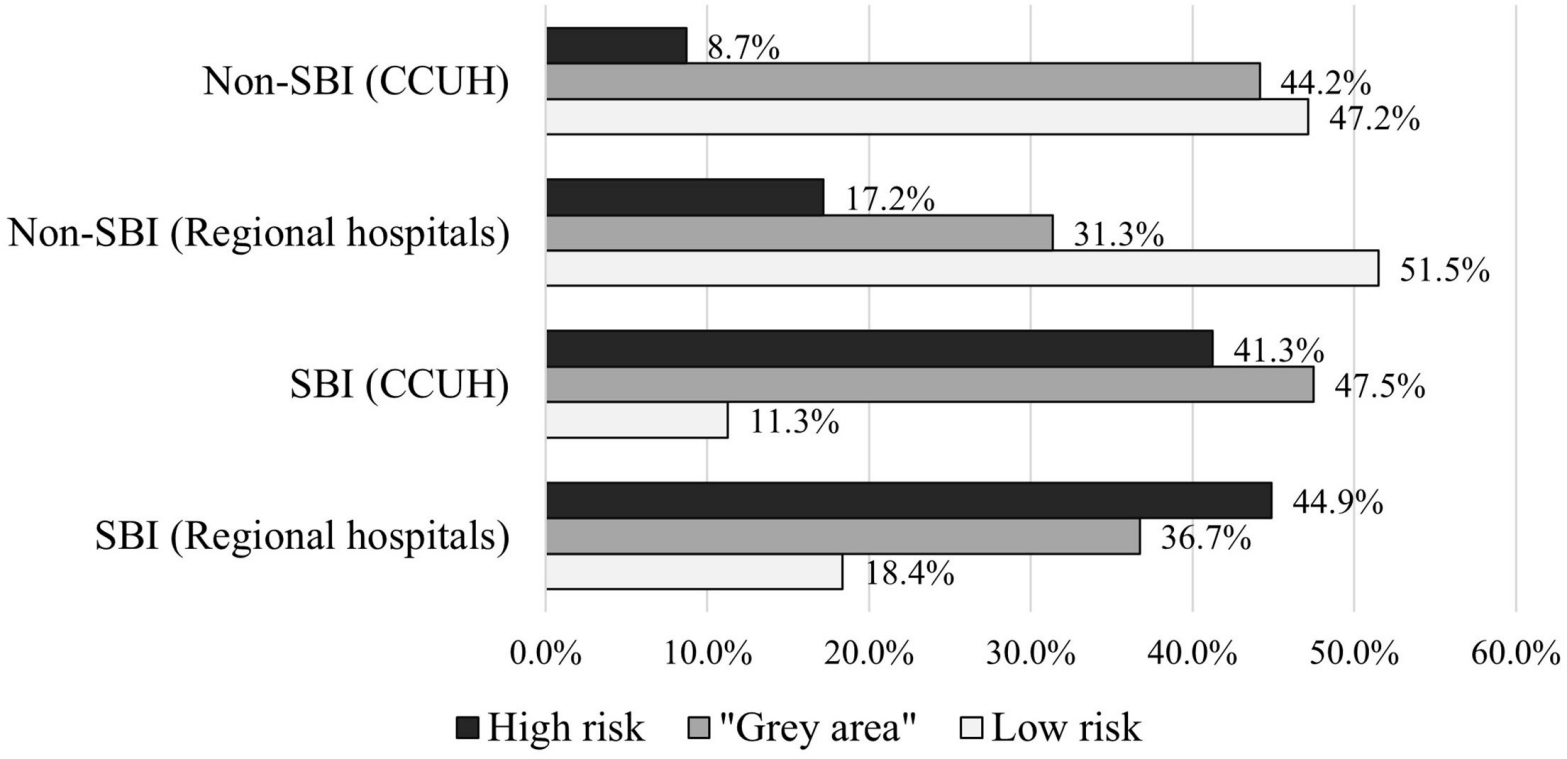
Categorization of patients with and without serious bacterial infection (SBI) in derivation and validation cohorts according to scoring system based on CPM 2.

**Figure 6:**
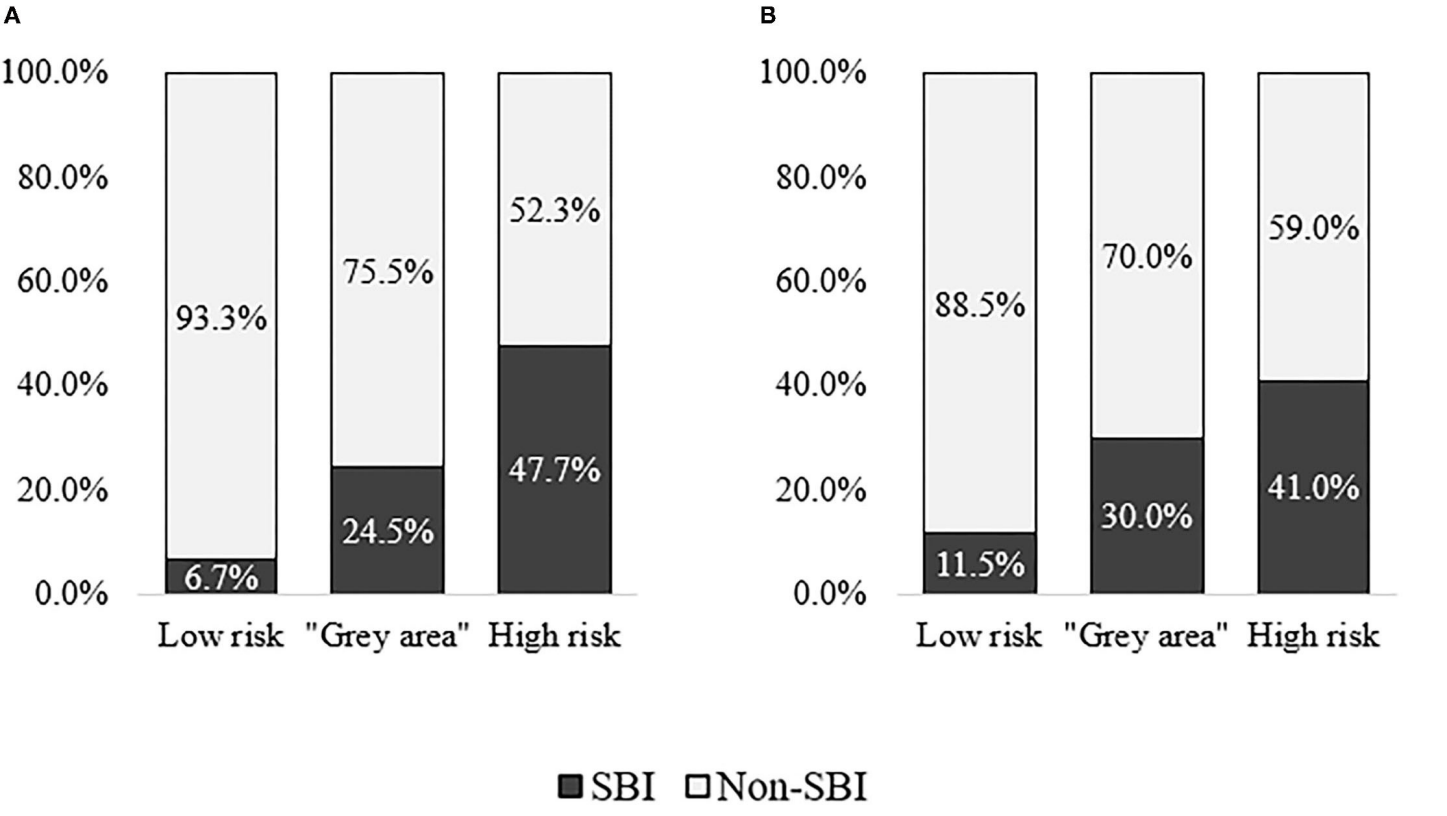
Composition of patients with and without serious bacterial infection (SBI) within low-risk, “gray area,” and high-risk categories in derivation and validation cohorts. **(A)** Derivation cohort (CCUH). **(B)** Validation cohort (Regional hospitals).

As a result, the majority of patients with SBI in the derivation cohort was categorized in either high risk or “gray area” categories, with the expense of missing 11.3% of SBI patients (*n* = 9). In validation cohort, 18.5% of patients with SBI (*n* = 9) were missed. Approximately half of the patients without SBI were categorized as low risk in both cohorts, while 8.7% (*n* = 23) and 17.2% (*n* = 23) of non-SBI patients were assessed as high-risk in derivation and validation cohorts, respectively. More details on categorization of patients in each cohort can be found in Supplementary Table 2. Figure 5 illustrates the distribution of patients with and without SBI between the different risk categories in derivation and validation cohorts. The composition of low-risk, “gray area,” and high-risk categories in each cohort is shown in Figure 6.

## Discussion

The CPMs derived in the study had moderate ability to predict SBI in febrile children presenting to ED. The performance of CPM 2, which included the clinician's intuitive “gut feeling” and “sense of reassurance,” was superior to CPM 1, which was based on clinical features alone. At a cut-off with the highest possible sensitivity and specificity set by Youden index, CPM 2 could accurately predict the outcome in more than three quarters of cases. Both models showed slight but acceptable decrease in performance in validation population.

Application of both models to derivation and validation populations still resulted in an overlap of patients with and without SBI near the cut-off with the highest possible sensitivity and specificity. Therefore, a scoring system from CPM 2, the superior model, was derived, leading to better identification of patients in the “gray area” and reduced the number of patients who would otherwise be segregated into a low-risk category.

### Comparison With Previous Studies

Several clinical prediction models for the recognition of SBI in febrile children have been proposed. The models with the most accurate ability to distinguish between SBI and non-SBI and with the best performance in validation studies are those containing laboratory markers in addition to clinical signs and symptoms (11, 25, 27, 29, 30, 52, 53). Not surprisingly, the diagnostic value of these prediction models was also superior to CPM 1 and CPM 2, which did not include laboratory variables. Nevertheless, the main goal of the study was to create a screening tool for selecting patients with increased risk of SBI and thus requiring further investigation. Therefore, we *a priori* decided to exclude any laboratory variables from our CPMs.

When compared to other prediction rules for serious infection in febrile children that are based on clinical parameters alone (7, 12, 13, 21), CPM 1 and CPM 2 show similar diagnostic performance in derivation cohort and better performance when prospectively validated externally. A clinical prediction rule for SBI in young children with fever without source presenting to ED developed by Bleeker et al. (7), including variables such as duration of fever, temperature above 40°C or below 36.7°C, vomiting, age above 1 year, chest wall retractions and/or tachypnoea, and poor peripheral circulation, and absence of poor micturition had an ROC area under the curve of 0.75 (0.68–0.83), which is similar to that of CPM 1 and CPM 2. However, the clinical model did not perform equally well when externally validated, yielding AUC of only 0.60 (0.49–0.70) in the validation cohort (52). An updated version including ill clinical appearance increased the AUC to 0.69 (0.63–0.75) in derivation population and to 0.65 (0.62–0.67) when validated in an external dataset, although in primary care (54). Similarly, another clinical score developed by Brent et al. (13) based on eight clinical variables showing moderate ability to predict SBI [AUC, 0.77 (0.71–0.83)] did not perform equally well when validated in external datasets (29).

There have also been attempts to validate prediction models derived from primary care to settings similar to ED, for example, decision tree developed by Van Den Bruel et al. (12), derived from a prospective study in primary care, including “gut feeling” that “something is wrong,” dyspnea, temperature above 39.95°C, diarrhea, and age, showed high sensitivity (96.8%) and specificity (88.5%). However, validation studies of the decision tree revealed poorer performance in ED settings (29, 55), with AUC ranging between 0.53 and 0.56 in febrile infants (29).

Our study did not show strong associations between tachycardia on admission and SBI as reported in other studies (9, 21). Similarly, temperature above 40°C did not increase the likelihood of SBI. Very high body temperature has been identified as one of the red flags in other prediction models (12, 21, 56), although in studies of populations with higher prevalence of SBI, it provides little diagnostic value (56). Surprisingly, refusal to drink in models derived from CCUH patients decreased the likelihood of SBI, which contradicts the findings of another study of febrile children presenting to ED in North of England (57), where poor feeding and restlessness were associated with increased risk for SBI. The study included patients with similar age range (0–16 years) but had a broader definition of serious illness, also including aseptic meningitis, and the study period excluded winter/spring months, which is the peak period for several viral illnesses such as influenza. It may be speculated that, as around half of febrile patients in CCUH are self-referred (44), this factor may have been one of the reasons for presenting to hospital even for a child with a self-limiting illness, due to availability of intravenous rehydration. However, the role of selection bias present in both derivation and validation cohorts should not be underestimated as a possible reason for these results, as parents of patients who remained at the ED for investigations and therapeutic intervention including intravenous rehydration were more likely to agree to participate in the study. The differences in variables associated/inversely associated with SBI indicate that further external validation in cohorts with consecutive enrolment, preferably in clinical settings in other countries, is necessary to evaluate the generalizability of CPM 1 and CPM 2.

Clinical impression of ill/toxic appearance was identified as the key variable in CPM 1. Strong association with ill appearance and serious illness has been found in studies in both primary care (35) and hospital EDs (11, 28, 52, 58). However, CPM 2, in which clinical impression was replaced by variables based on non-analytical and intuitive reasoning of the clinician—“gut feeling,” and “sense of reassurance,” had a higher diagnostic value when compared to CPM 1. Clinician's “sense of alarm” of “something wrong” has previously been integrated with clinical signs in a CPM designed by Van den Bruel et al. (12), in which it was the strongest predictive factor. Another primary care study found evidence of a high predictive value for clinician's “gut feeling” in recognizing serious illness (35), although the same diagnostic strength of this variable in ED settings has not been verified (43). The other type of intuitive reasoning, “sense of reassurance,” has been included in a prediction model for the first time in this study, where it has been identified as the strongest variable that decreases the probability of SBI in CPM 2.

### Strengths and Limitations

The main strengths of this study are prospective enrolment of both derivation and validation cohorts, and application of uniform case report forms, which enabled the researchers to collect information on all variables with trustworthy accuracy, without a necessity for proxy variables. There was a slight decrease in diagnostic performance of both CPM 1 and CPM 2 when applied to validation cohorts; however, a decrease in this magnitude can be expected and does not indicate overfitting of the model. The models had moderate ability to predict SBI in both derivation and validation cohorts, despite the fact that they were drawn from settings with different level of care (tertiary vs. secondary). It must be noted that patients at increased risk for infection due to comorbidities (who are more likely to present to tertiary care) were excluded.

The limitations of the study are the following. As informed consent from a parent or guardian was required for participation in the study, consecutive enrolment was not possible, and the study samples are relatively small. The large number of missing variables caused the number of complete cases to be lower than a preferred sample size. No data were imputed to replace the missing variables, as “gut feeling” and “sense of reassurance” are based on the intuitive and non-analytical interpretation of the clinical situation by the doctor; thus, replacing the missing values with software-generated imputations was considered as inappropriate. The prevalence of SBI though is similar in the cohorts used to develop both models, and the performance of CPM 2 in an independent validation cohort is close to that in the derivation cohort.

A selection bias toward sicker children is evident due to requirement by the PERFORM project to collect blood samples for purposes not related to this particular study and because parents spending longer time at the ED in CCUH and regional hospitals alike were more likely to provide informed consent and ensure participation of parents in the questionnaire on parental concern. The selection bias is reflected by the high prevalence of SBI in both cohorts, which exceeds that reported in similar settings elsewhere in developed countries. The high prevalence of the outcome of interest may have affected the performance rates of the CPMs in the biased samples. This raises concerns about the applicability of the models on general population of febrile children presenting to ED, where the expected prevalence of SBI is lower. Therefore, efficacy of the models in recognition of SBI in all febrile patients presenting to pediatric ED needs to be evaluated by application to patient cohorts with consecutive enrolment.

Only half of the derivation cohort consists of children younger than 5 years, which is possibly due to requirement of additional blood samples for the PERFORM project, a factor that may have influenced parental decision to participate in the study. This is the age group in which early diagnosis of SBI is the most challenging and important, and applicability of the models to this study population might be affected by the low numbers in the derivation cohort. However, the proportion of children under 5 was significantly higher in validation cohort (81.4%), and the performance of both models, especially CPM2, was close to that in the derivation cohort.

A significant proportion of patients in both cohorts were lost to telephone follow-up, and the outcome of illness was based on data available in hospital databases, the determining factor being readmission to hospital as per the stated definition of SBI. While hospital readmission of patients first assessed in regional hospitals was ruled out at the same hospital and CCUH as the reference hospital, hospitalizations to other regional hospitals were not researched, nor were admission of patients previously discharged from CCUH to any of the regional hospitals. Although the likelihood of these given hospital admissions is very low due to the nature of healthcare system in Latvia, this leaves a theoretical possibility that a readmission of a patient categorized as non-SBI may have been missed.

The level of experience of the clinician was not taken into account due to complexity in inclusion of such variable in a CPM, although a previously published study reports that the diagnostic value of “gut feeling” and “sense of reassurance” is higher when expressed by senior clinicians as opposed to medical residents (42).

The main outcome of the study was the presence of SBI, which implies that non-bacterial serious illnesses such as aseptic meningitis, viral gastroenteritis with dehydration, and severe bronchiolitis with respiratory insufficiency were classified as non-SBI, together with other, milder illnesses. This was done due to prioritizing screening for patients who might benefit from early initiation of antimicrobial treatment, while the treatment for the viral serious illnesses is mostly symptomatic. However, it also means that the model cannot be applied for screening of all serious illnesses.

Though recognizing SBI in the majority of patients, the assessment score based on CPM2 still missed a significant proportion of patients with SBI in validation population. This indicates that patients who are categorized as low risk according to the score should still be approached with caution, and additional tools such as thorough clinical examination for clinical “red flag” signs not included in the model should be used.

The heterogeneity of the main outcomes of the study (presence or absence of SBI) is another limitation of this study, although it is shared with other studies on recognition of serious illness in febrile/acutely ill children. The infections included in the selected definitions of SBI affect different organ systems and could manifest with a large spectrum of signs and symptoms, some more typical in one condition than in another, thus selection of clinical variables that are useful for identification of all SBIs may be perceived as unreasonable. On the other hand, focusing on ruling out each one of the outcomes separately is contradictory to the main purpose of this study, which was to create a single, easily applicable screening model for further guidance in management of a wide range of patients presenting to ED with fever. It must be noted though that splitting the outcomes into different subtype categories of SBI, such as pneumonia, urinary tract infections, bacteremia, and others, may have resulted in higher diagnostic accuracy (8, 11).

### Clinical and Research Implications

This study introduces CPM 1 and CPM 2 as externally validated tools to aid pediatricians and pediatric residents in initial assessment of febrile children presenting to emergency departments. It must be noted, however, that due to the biased samples in both derivation and validation cohorts in this study, more validation studies are required before implementation of the models in clinical practice.

Like other prediction models, for example, Feverkidstool (11), the CPMs derived in this study may help to recognize patients with a high probability of SBI and, with the aid of the scoring system based on CPM2, to identify patients who are in the uncertain “gray area,” in which SBI and non-SBI are equally likely. This may be especially useful in directing a more purposeful investigation process and administration of antibacterial therapy in cases when patients present at early stages of illness, when any “red flag” signs for a specific illness may be absent. The advantage of CPM1 and CPM2 is that no laboratory values are required for the risk assessment, which is convenient for settings with high flow of patients where rapid point-of-care tests are unavailable.

As a high proportion of patients classified as “high risk” according to the scoring system based on CPM 2 were diagnosed with SBI, we propose that, for patients who fall into this category, early antibacterial therapy should be considered. In case of long expected waiting time for investigation results, antimicrobial treatment may be administered before these results become available, especially in cases that present with alarming signs and symptoms for invasive bacterial infection, sepsis, or septic shock, according to other widely used screening tools. Although only 47.7% and 41.0% of patients in this category in derivation and validation cohorts, respectively, were diagnosed with SBI, studies on screening tools such as NICE Sepsis guidelines reveal even lower percentage of the outcome of interest in patients whose clinical presentation indicates the necessity for early antimicrobial intervention (6). The recommended maximum delay time for antimicrobial therapy varies among guidelines in children with suspected sepsis, from 1 h in children with high-risk signs for sepsis (47) to 3 h in potentially septic children without signs of shock (59). In this study, though, the outcome of interest was SBI instead of sepsis, invasive bacterial infection, or septic shock; therefore, it is not clear if the same level of caution is applicable. If, however, the clinician decides to initiate early antimicrobial treatment for patients in “high-risk” category, it is possible and necessary to de-escalate antimicrobial treatment if the diagnosis of SBI is not confirmed.

Approximately one-third of patients with SBI fell in the “gray area”; therefore, additional diagnostic interventions such as laboratory tests, diagnostic imaging, and/or repeated clinical assessment at a later stage of the disease should be performed to clarify the diagnosis in patients who are classified in this category, while “watchful waiting” could be applied to patients whose assessed risk for SBI is low. The CPMs do not overrule any guidelines for assessment and management of febrile patients in pediatric settings. Other signs and symptoms associated with SBI and listed as “red” features in NICE “Traffic light system for identifying risk for serious illness” but not included in the CPMs due to low incidence in research population, such as cyanosis, petechial rash, meningeal signs, or focal seizures (18, 20), should also be considered.

This study adds to understanding of how clinician's subjective review together with clinical signs can improve recognition of serious illness in pediatric emergency department. CPM 2 is so far the first prediction rule for SBI in febrile patients presenting to ED to include variables based on clinician's non-analytical reasoning. Another example is Paediatric Observation Priority Score (POPS), a triage tool based on physiological signs and clinician's gut feeling intended for the assessment of severity of a child's condition and need for specialist review/admission when presenting to healthcare with acute illness of infectious or non-infectious origin (60–62).

Although specialists tend to be cautious with relying on their intuitive feelings in medical practice, the role of intuition in diagnostic reasoning has been recognized by clinicians working in general practice and hospitals alike, especially in scenarios with little time for analytic reasoning (63–66). “Sense of alarm,” a term similar to “gut feeling” used in this study, is a recognized intuitive feeling that leads to closer evaluation and investigation and has been regarded as valuable source of judgement (63, 64), while the approach to “sense of reassurance” seems a bit more cautious, suggesting that the clinician should still be on their guard not to underestimate the situation (64). This study reveals “sense of reassurance” as the strongest variable to rule out SBI, and the non-analytic part of assessment is balanced by assessment of objective signs and symptoms in CPM 2. Due to the superior performance in validation population, it is evident that the intuitive part of assessment enhances the analytical reasoning of the clinician. Therefore, we suggest that, during clinical evaluation of the patient, clinicians should examine their intuitive feelings and consider them when deciding on the management of each case.

Both CPMs developed in this study have so far only been validated in a small population of patients presenting to the EDs in hospitals of the same country. External validation in EDs in different countries, preferably in large patient populations with consecutive enrolment, and in settings with lower prevalence of SBIs, such as secondary or primary care, should be performed for reliable assessment of the applicability of the models to various patient populations.

## Data Availability Statement

The original contributions presented in the study are included in the article/Supplementary Material, further inquiries can be directed to the corresponding author/s.

## Ethics Statement

Enrolment of CCUH patients in the PERFORM (Personalised Risk Assessment in Febrile Illness to Optimise Real-Life Management Across the European Union) project was approved by the Central Medical Ethics Committee of the Republic of Latvia (Decision No. 1/16-07-14; approval date 26.05.2016). The inclusion of additional cohort of CCUH patients was approved by the Ethics Committee of Riga Stradins University (Decision No. 13/05.10.2017), which also approved of enrolment of patients of regional hospitals in the validation cohort (Decision No. 6-3/27, approval date 25.10.2018), after obtaining consent for the study from the Institutional Review Board of Children's Clinical University Hospital, as well as from the designated officials in the regional hospitals. Written informed consent to participate in this study was provided by the participants' legal guardian/next of kin.

## Author Contributions

UU contributed to conceptualization, methodology (study design and protocol, design of the data collection instruments), data collection, data curation, analysis, interpretation, visualization (development of tables and figures), and writing—preparation of the original draft. EP contributed to methodology (methods for data collection, statistical analysis, variable selection, and derivation of the prediction models), data curation and analysis, visualization (development of tables and figures), and writing—preparation of the original draft. DZ revised the methodology (study design and protocol, design of the data collection instruments), coordinated and supervised patient enrolment and data collection, analysis, interpretation, and revised and edited the original draft of the manuscript. JP conceptualized and designed the study, designed the data collection instruments, coordinated and supervised data collection, analysis, interpretation, and critically reviewed and edited the original draft of the manuscript. All authors have read the final version of the manuscript and agreed to its submission.

## Funding

The study was partially derived from the PERFORM (Personalised Risk Assessment in Febrile Illness to Optimise Real-Life Management Across the European Union) project, which has been supported by funding received from the European Union's Horizon 2020 research and innovation program (PERFORM) under Grant Agreement No. 668303.

## Conflict of Interest

The authors declare that the research was conducted in the absence of any commercial or financial relationships that could be construed as a potential conflict of interest.

## Publisher's Note

All claims expressed in this article are solely those of the authors and do not necessarily represent those of their affiliated organizations, or those of the publisher, the editors and the reviewers. Any product that may be evaluated in this article, or claim that may be made by its manufacturer, is not guaranteed or endorsed by the publisher.
