# Supplementary material for: Integrating Clinical Signs at Presentation and Clinician's Non-analytical Reasoning in Prediction Models for Serious Bacterial Infection in Febrile Children Presenting to Emergency Department
Source: Front Pediatr. 2022 Apr 25;10:786795. doi: 10.3389/fped.2022.786795 (PMC9082163; doi:10.3389/fped.2022.786795)
Supplement: Supplementary file 1 [file Data_Sheet_1.PDF]

Supplementary file 1 **Research No. of the patient ___________**

Questionnaire of the view of parents/guardians on their child's illness

Dear parents / guardians,

We are very grateful for your participation in this questionnaire. The purpose of this questionnaire is to clarify your observations and feelings concerning febrile illness in your child. By gathering your answers and those of other participants, we aim to assess the value of the information provided by parents in early recognition of serious infections in children, so that parental opinion could be taken into consideration to a greater extent when evaluating children with fever in future. The completion of the questionnaire will not take longer than 15 minutes.

The survey will include questions regarding the ongoing episode of your child's illness, as well as questions on your general beliefs about fever in children.

Your consent or refusal to participate in this questionnaire will affect neither the management of your child's illness in the hospital nor the attitude of the healthcare personnel towards you or your child. You have the right to refuse further participation at any moment, as well as to demand the withdrawal of already given data from being analysed for the study. In that case, please inform any of the healthcare personnel, or the research team about your decision.

Participant of the survey (circle the appropriate) Mother, Father, Other________________

No. of children in the family / household: _________________

Order of birth (first / second/ third**) of the child that is our patient ___________**

Age of the mother (carer): ______________

Level of education of the mother (carer) (circle the appropriate)

1) Middle school
2) High school
3) Professional ______________________
4) Incomplete higher education
5) Higher education (level of degree): ___________________________
6) Other ____________________________

Age of the father (carer): ______________

Level of education of the father (carer) (circle the appropriate)

1) Middle school
2) High school
3) Professional ______________________
4) Incomplete higher education
5) Higher education (level of degree): ___________________________
6) Other ____________________________

Supplementary file 1 **Research No. of the patient ___________**

1. **How many times has your child been ill over the last 12 months?**

2. **How many times over the last 12 months have you sought help from your family doctor due to increased body temperature of this child? _____________**

3. **How many times has your child been hospitalized for longer than 24 hours? ______________________**

4. **Has your child previously had any of the following infectious diseases, during which antibiotics were prescribed? (Mark the appropriate with X)**

	Once	Repeatedly
1) The child has had none of these infections	☐	☐
2) Sinusitis	☐	☐
3) Tonsillitis with use of antibiotics	☐	☐
4) Pneumonia	☐	☐
5) Bronchitis with use of antibiotics	☐	☐
6) Urinary tract infection	☐	☐
7) Gastrointestinal infection with use of antibiotics	☐	☐
8) Bacterial meningitis	☐	☐
9) Acute osteomyelitis	☐	☐
10) Septic arthritis	☐	☐
11) Sepsis	☐	☐
12) Other illness with use of antibiotics _________	☐	☐

5. **Have you observed any of the following in your child during this episode of illness?**

1) The child was breathing shallower or faster ☐
2) The child was grunting/ moaning ☐
3) The child had a changed skin color (greyish / pale) ☐
4) The child was unwilling to play with his/her favourite toys ☐
5) The child was crying the whole time and it was hard to calm him/her down ☐
6) The child had an atypical cry ☐
7) The child was screaming ☐

8) The child was irritated and restless ☐
9) The child slept longer than normally, was very sleepy ☐
10) The child was eating less or refused food ☐
11) The child was drinking less or refused to drink ☐
12) The child had decreased urination ☐
13) The child's urine had an unusual smell ☐
14) Other observed changes ______________________________ ☐

6. Did the child feel better after you gave him/her medication to reduce the temperature? (Choose one)

1) Yes, the child became active as usual ☐
2) The child felt better but his behaviour was still not as usal ☐
3) The child did not feel better ☐
4) The temperature did not go down ☐
5) The child got worse and worse ☐
6) I did not give my child such medication. ☐

7. When this episode if child's illness started, did you have a feeling that this time is different/ more severe than other times when your child has had fever? (Choose one)

1) Definitely yes ☐
2) Most likely yes ☐
3) More likely yes than no ☐
4) Difficult to say ☐
5) More likely no than yes ☐
6) Most likely no ☐
7) Definitely no ☐

8. Did you have a feeling that this tie your child needed medical help more urgently than other times when she/he has had a fever?

1) Yes ☐
2) No ☐
3) Difficult to say ☐

9. **For how long had your child been ill before you sought medical help for the first time?**
   1) 0-6 hours ☐
   2) 6-12 hours ☐
   3) 12-24 hours ☐
   4) 24-48 hours (2 days) ☐
   5) 48-72 hours (3 days) ☐
   6) Longer _______________ ☐

10. **Day of the week (***for example, Sunday***) , when your child got ill: ____________________**

11. **Day of the week and time when you first sought help for your child (day, hh:mm)**

    ____

12. **How would you evaluate your level of concern when your child got ill this time?**
    1) I was very concerned, unlike any other time ☐
    2) I was concerned more than other times when she/ he has been ill ☐
    3) I was not concerned more than other times when she/ he has been ill ☐
    4) I was concerned not as much as other times when she/ he has been ill ☐
    5) I was not concerned at all ☐

13. **Where did you seek help first during this episode of the child's illness?**
    1) Family doctor's appointment ☐
    2) Consultation over the phone ☐
    3) Out-of-hours healthcare service ☐
    4) Ambulance ☐
    5) Hospital ☐
    6) Other ____________________________ ☐

14. **Did the healthcare professional mentioned above provide a sufficient explanation of what was going on and of the reasons for the fever?**
    1) Yes ☐
    2) No ☐
    3) Partially ☐

15. Did the conversation with the healthcare professional mentioned above help to reduce your anxiety about your child's illness?

1) Yes ☐
2) My anxiety did not change ☐
3) My anxiety increased ☐

16. Did the healthcare professional you were seen by at this hospital sufficient explanation of what was going on and of the reasons for the fever?

4) Yes ☐
5) No ☐
6) Partially ☐

17. Did the conversation with the healthcare professional you were seen by in this hospital help to reduce your anxiety about your child's illness?

4) Yes ☐
5) My anxiety did not change ☐
6) My anxiety increased ☐

Thank you for your answers on this illness of your child! From this point on we would like to ask you about your beliefs on the management of fever in general.

18. What is a very high temperature, in your opinion? ________________ °C

19. At what temperature would you give your child medication to reduce it? Above........ °**C**

20. What medication would you give your child to reduce fever?

Ibuprofen (Nurofen, Ibustar, Ibumetin, Ibufen) ☐
Paracetamol (Panadol, Efferalgan, calpol) ☐
Other (which one........................) ☐

21. How would you choose the dosage of medication?

1) As the doctor recommended ☐
2) As the packaging says ☐
3) Whatever I feel like, depends on the temperature ☐
4) Other ☐

22. In your opinion, is there such thing as a dangerous level of body temperature?

1) Yes (Above.......... °**C**) ☐
2) No ☐
3) I don't know ☐

23. IS fever itself a sign of a serious and potentially dangerous illness?

1) yes ☐
2) No ☐
3) Other symptoms must be present as well ☐
4) I don't know ☐

24. How soon after any of your children develops fever would you seek for medical help?

1) 0-6 hours ☐
2) 6-12 hours ☐
3) 12-24 hours ☐
4) 24-48 hours (2nd day) ☐
5) 48-72 hours (3rd day) ☐
6) Later (when) ________________ ☐

25. Does being treated in the hospital setting give you a better feeling of safety than care at home under supervision of your family doctor?

1) Yes ☐
2) No ☐
3) Partially ☐

26. How would you evaluate the availability of your family doctor?

1) Very good ☐
2) Good ☐
3) More likely good than bad ☐
4) Normal ☐
5) More likely bad than good ☐
6) Bad ☐
7) Very bad ☐
