# Supplementary material for: Integrating Clinical Signs at Presentation and Clinician's Non-analytical Reasoning in Prediction Models for Serious Bacterial Infection in Febrile Children Presenting to Emergency Department
Source: Front Pediatr. 2022 Apr 25;10:786795. doi: 10.3389/fped.2022.786795 (PMC9082163; doi:10.3389/fped.2022.786795)
Supplement: Supplementary file 2 [file Data_Sheet_2.PDF]

Supplementary file 2 **Pacienta identifikācijas numurs ________**

Vecāku / aizbildņu aptauja par bērna saslimšanu

Cienījamie vecāki / aizbildņi!

Izsakām Jums lielu pateicību par piedalīšanos šajā aptaujā. Šīs aptaujas mērķis ir noskaidrot Jūsu novērojumus un izjūtas par Jūsu bērna saslimšanu. Apkopojot Jūsu un citu bērnu vecāku atbildes, paredzēts izzināt vecāku sniegtās informācijas vērtību agrīnā smagu infekciju atpazīšanā bērniem, lai nākotnē palielinātu vecāku lomu šo infekciju agrīnā diagnostikā un ārstēšanā bērniem. Aptaujas aizpildīšana aizņems aptuveni 15 minūtes.

Pēc šīs aptaujas, ja tam piekritīsiet, būsim ļoti pateicīgi, ja piedalīsieties detalizētākā sarunā (ilgums aptuveni 15 - 30 minūtes) ar pētniecības komandas pārstāvi, kuras laikā Jums tiks uzdoti plašāki jautājumi par Jūsu izjūtām un novērojumiem sakarā ar šo sava bērna saslimšanas reizi. Sarunas laikā tiks veikts tās audioieraksts, kas būs anonīms (identifikācijā tiks izmantots Jūsu bērna reģistrācijas numurs pētījumā). Intervijas saturs būs zināms tikai pētniecības komandai un netiks atklāts trešajām personām.

Jūsu piekrišana vai atteikšanās piedalīties šajā aptaujā un detalizētākajā sarunā neietekmēs Jūsu bērna ārstēšanas procesu vai attiecības ar bērna ārstēšanā iesaistīto medicīnisko personālu. Jums ir tiesības jebkurā mirklī pārtraukt dalību aptaujā vai sarunā, kā arī atteikties no sniegto datu izmantošanas pētījumā, šādā gadījumā informējot ārstniecības personālu vai kādu no pētniecības komandas locekļiem.

Aptaujā piedalās (vajadzīgo apvilkt): Māte, Tēvs, Cits________________

Bērnu skaits ģimenē: _________________

Kurš pēc kārtas (pirmais / otrais/ trešais**) jūsu ģimenē ir pašreiz saslimušais bērns?** ___________

Bērna mātes (aizbildnes) vecums gados: ______________

Bērna mātes (aizbildnes) izglītības līmenis (vajadzīgo pasvītrot):

1) Pamata
2) Vidējā
3) Profesionālā (kāda)______________________
4) Nepabeigta augstākā
5) Augstākā (grāds): ___________________________
6) Cita (kāda) ____________________________

Bērna tēva (aizbildņa) vecums gados: ______________

Bērna tēva (aizbildņa) izglītības līmenis (vajadzīgo pasvītrot):

1) Pamata
2) Vidējā
3) Profesionālā (kāda)______________________
4) Nepabeigta augstākā
5) Augstākā (grāds): ___________________________
6) Cita (kāda) ___________________________

Supplementary file 2 **Pacienta identifikācijas numurs ________**

1. **Cik bieži jūsu bērns ir slimojis pēdējo 12 mēnešu laikā? _________________**

2. **Cik bieži pēdējo 12 mēnešu laikā esat vērsušies pēc palīdzības pie ārsta sakarā ar to, ka bērnam bijusi paaugstināta temperatūra? ____________**

3. **Cik reizes dzīves laikā Jūsu bērns bijis stacionēts slimnīcā ilgāk par 24 stundām? _____________________**

4. **Vai jūsu bērnam iepriekš bijušas kādas no sekojošām infekcijas saslimšanām, kuru laikā lietotas antibiotikas? (atbilstošos variantus atzīmēt ar X)**

	Vienreiz	Atkārtoti
1) Bērnam nav bijušas šādas infekcijas	☐	☐
2) Deguna blakusdobumu iekaisums	☐	☐
3) Angīna, kuras ārstēšanā lietotas antibiotikas	☐	☐
4) Plaušu karsonis	☐	☐
5) Bronhīts, kura ārstēšanā lietotas antibiotikas	☐	☐
6) Urīnceļu infekcija	☐	☐
7) Kuņģa un zarnu trakta saslimšana, kuras ārstēšanai lietotas antibiotikas	☐	☐
8) Bakteriāls meningīts	☐	☐
9) Akūts osteomielīts	☐	☐
10) Septisks artrīts	☐	☐
11) Sepse	☐	☐
12) Cita saslimšana (kāda), kuras ārstēšanā lietotas antibiotikas _________	☐	☐

5. **Vai šajā bērna saslimšanas reizē esat novērojis/usi kādu no šīm pazīmēm?**

1) Bērns elpo seklāk vai biežāk ☐
2) Bērns sten, vaid ☐
3) Bērnam ir izmainīta ādas krāsa (pelēcīga/bāla) ☐
4) Bērns atsakās no iemīļotajām aktivitātēm un rotaļlietām ☐
5) Bērns ir raudulīgs, grūti nomierināms ☐
6) Bērnam ir izmainīts raudāšanas veids ☐
7) Bērns kliedz ☐

8) Bērns izteikti satraukts un uzbudināts ☐
9) Bērns guļ vairāk nekā parasti, ir miegains ☐
10) Bērns mazāk ēd vai atsakās no ēdiena ☐
11) Bērns mazāk dzer vai atsakās no dzēriena ☐
12) Bērnam samazināts urīna daudzums ☐
13) Bērnam izmainīta urīna smarža ☐
14) Citas īpašas pazīmes (kādas)______________________________ ☐

6. Vai tad, kad bērnam iedevāt temperatūru pazeminošus līdzekļus, bērna pašsajūta uzlabojās (atzīmēt vienu)?

1) Jā, bērns kļuva aktīvs kā ierasts ☐
2) Bērna pašsajūta uzlabojās, bet saglabājās izmainīta uzvedība ☐
3) Bērna pašsajūta neuzlabojās ☐
4) Temperatūra nemazinājās ☐
5) Bērnam palika arvien sliktāk ☐
6) Temperatūru pazeminošus līdzekļus bērnam nedevu. ☐

7. Vai, sākoties pašreizējai saslimšanai, Jums bija sajūta, ka šoreiz bērns saslimis smagāk kā iepriekšējās reizes (atzīmēt vienu)?

1) Noteikti jā ☐
2) Visticamāk jā ☐
3) Drīzāk jā nekā nē ☐
4) Grūti pateikt ☐
5) Drīzāk nē nekā jā ☐
6) Visticamāk nē ☐
7) Noteikti nē ☐

8. Vai Jums bija sajūta, ka šoreiz bērnam medicīniskā palīdzība nepieciešama steidzamāk kā citas reizes, kad bērns slimojis ar paaugstinātu temperatūru?

1) Jā ☐
2) Nē ☐
3) Grūti pateikt ☐

9. Cik ilgi Jūsu bērnam jau bija slimības pazīmes, pirms meklējāt medicīnisko palīdzību?

1) 0-6 stundas ☐
2) 6-12 stundas ☐
3) 12-24 stundas ☐
4) 24-48 stundas (2. diennakts) ☐
5) 48-72 stundas (3. diennakts) ☐
6) Ilgāk (cik) _______________ ☐

10. Nedēļas diena (*piem. svētdiena*) , kad bērns saslima: _________________________

11. Nedēļas diena un laiks (hh:mm) (*piem. pirmdiena, 13:00*), kad pirmo reizi meklējāt palīdzību _________________

12. Kā Jūs vērtējat savu satraukumu par bērna saslimšanu?

1) Biju ļoti satraukts/ta, kā nekad agrāk ☐
2) Biju satraukts/ ta vairāk kā citas reizes, kad bērns slimojis ☐
3) Nebiju satraukts/ta vairāk kā citas reizes, kad bērns slimojis ☐
4) Biju satraukts/ ta mazāk kā citas reizes, kad bērns slimojis ☐
5) Nebiju satraukts/ta nemaz ☐

13. Pie kā vērsāties pēc medicīniskās palīdzības pirmo reizi sakarā ar šo sava bērna saslimšanas reizi?

1) Ģimenes ārsts ☐
2) Ģimenes ārstu konsultatīvais tālrunis ☐
3) Rajona dežūrārsts ☐
4) Ātrā palīdzība ☐
5) Slimnīca ☐
6) Cits _____________________________ ☐

14. Vai no iepriekš minētā medicīnas darbinieka saņēmāt pietiekamu izskaidrojumu par visu notiekošo, par paaugstinātas temperatūras iemesliem?

1) Jā ☐
2) Nē ☐
3) Daļēji ☐

15. Vai Jūsu satraukums par bērna saslimšanu mazinājās pēc sarunas ar iepriekš minēto medicīnas darbinieku?

1) Jā ☐
2) Satraukums nemainījās ☐
3) Satraukums pieauga ☐

16. Vai no Bērnu klīniskās universitātes slimnīcas medicīnas personāla saņēmāt pietiekamu izskaidrojumu par visu notiekošo, par paaugstinātas temperatūras iemesliem?

1) Jā ☐
2) Nē ☐
3) Daļēji ☐

17. Vai Jūsu satraukums par bērna saslimšanu mazinājās pēc tam, kad bērnu apskatīja Bērnu klīniskās universitātes slimnīcas ārsti?

1) Jā ☐
2) Satraukums nemainījās ☐
3) Satraukums pieauga ☐

Pateicamies par Jūsu atbildēm par bērna saslimšanu! Tālāk vēlamies izzināt Jūsu uzskatus par ārstēšanas un aprūpes taktiku gadījumā, ja bērns slimo ar paaugstinātu temperatūru!

18. Kāda, pēc Jūsu domām, ir ļoti augsta temperatūra? ________________ °C

19. Pie kāda temperatūras pacēluma Jūs saviem bērniem dodat temperatūru pazeminošos līdzekļus? Virs............ °**C**

20. Kādus medikamentus Jūs dodat saviem bērniem, lai samazinātu temperatūru?

Ibuprofēns ☐
Paracetamols ☐
Cits (lūdzu ierakstiet....................... °**C**) ☐

21. Cik lielu medikamenta devu Jūs dodat saviem bērniem, lai samazinātu temperatūru?

1) Kā ārsts rekomendējis ☐
2) Kā rakstīts uz iepakojuma ☐
3) Pēc sajūtām atkarībā no temperatūras ☐
4) Cits ☐

22. Vai, jūsuprāt, eksistē bīstams temperatūras pacēlums?

1) Jā (Virs.......... °**C**) ☐
2) Nē ☐
3) Nezinu ☐

23. Vai paaugstināta temperatūra pati par sevi norāda uz bīstamu un nopietnu saslimšanu?

1) Jā ☐
2) Nē ☐
3) Jābūt vēl citiem simptomiem ☐
4) Nezinu ☐

24. Cik ilgi pēc temperatūras paaugstināšanās saviem bērniem jūs parasti meklējat medicīnisko palīdzību?

1) 0-6 stundas ☐
2) 6-12 stundas ☐
3) 12-24 stundas ☐
4) 24-48 stundas (2. diennakts) ☐
5) 48-72 stundas (3. diennakts) ☐
6) Vēlāk (kad) _______________ ☐

25. Vai atrašanās stacionārā Jums dod lielāku drošības sajūtu kā ārstēšanās ģimenes ārsta uzraudzībā?

1) Jā ☐
2) Nē ☐
3) Daļēji ☐

26. Kā Jūs vērtējat sava ģimenes ārsta pieejamību?

1) Ļoti laba ☐
2) Laba ☐
3) Vairāk laba nekā slikta ☐
4) Normāla ☐
5) Vairāk slikta nekā laba ☐
6) Slikta (minēt iemeslu) ___________________ ☐
7) Ļoti slikta (minēt iemeslu) ________________ ☐
