# Supplementary material for: Integrating Clinical Signs at Presentation and Clinician's Non-analytical Reasoning in Prediction Models for Serious Bacterial Infection in Febrile Children Presenting to Emergency Department
Source: Front Pediatr. 2022 Apr 25;10:786795. doi: 10.3389/fped.2022.786795 (PMC9082163; doi:10.3389/fped.2022.786795)
Supplement: Supplementary file 3 [file Data_Sheet_3.PDF]

Supplementary file 3 **Research code of the patient ________**

Diagnostic value of clinical presentation, parental concern and clinicians' gut feeling in identifying serious bacterial infections in febrile children.

Clinician's questionnaire

1. What is your evaluation of the overall condition of the child after initial examination?

    a) Mild illness / normal
    b) Moderate
    c) Severe
    d) Critical / life threatening

2. After the examination of the child, do you have an impression / intuitive feeling that the child has a serious illness?
    a) Yes
        For what reason:
        i. Am able to explain_______________________________________
        ii. Am not able to explain
    b) The possibility cannot be excluded
    c) No

3. After the examination of the child, do you have an impression / intuitive feeling that the child has a mild or self – limiting illness?
    a. Yes
        For what reason:
        1. Am able to explain_______________________________________
        2. Am not able to explain
    b. Am not sure
    c. No

4. Based on the examination data, circle the possible primary diagnoses:

    a) Skin and soft tissue infection
    b) Urinary tract infection
    c) Pneumonia
    d) Bacterial gastroenteritis
    e) Bacterial meningitis
    f) Acute osteomyelitis
    g) Purulent arthritis
    h) Bacterial infection of unspecified site
    i) Sepsis
    j) None of the above

Supplementary file 3 **Research code of the patient ________**

5. Which of the following features are present in the child's physical examination data or history of this episode? (Circle the appropriate)

    a) Ill appearance
    b) Lethargy / drowsiness
    c) Grunting
    d) Inconsolable crying
    e) Cyanosis
    f) Tachypnoea
    g) Shortness of breath
    h) Poor peripheral perfusion
    i) Positive meningeal signs
    j) Non-blanching rash / petechiae
    k) Seizures
    l) Hypotension
    m) Unconsciousness

7. Respondent data:
    a) Licensed doctor: work experience as a doctor (years) ______________________
    b) Medical resident: year of training ______________
