# Supplementary material for: Integrating Clinical Signs at Presentation and Clinician's Non-analytical Reasoning in Prediction Models for Serious Bacterial Infection in Febrile Children Presenting to Emergency Department
Source: Front Pediatr. 2022 Apr 25;10:786795. doi: 10.3389/fped.2022.786795 (PMC9082163; doi:10.3389/fped.2022.786795)
Supplement: Supplementary file 4 [file Data_Sheet_4.PDF]

Supplementary file 4 **Pacienta identifikācijas Nr __________**

Klīnisko pazīmju kopuma, vecāku un ārstu izvērtējuma nozīme bērnu ar drudzi izmeklēšanā un ārstēšanā, kā arī smagu bakteriālu infekciju agrīnā atpazīšanā.

Ārsta anketa

1. Kāds ir jūsu vērtējums par bērna vispārējo stāvokli pēc pirmās apskates?

   a) Viegls
   b) Vidēji smags
   c) Smags
   d) Ļoti smags/kritisks
2. Vai pēc bērna pirmās apskates jums palika iespaids / intuitīva sajūta, ka bērnam ir smaga saslimšana?
   a) Jā
      Kāpēc:
      i. Varu percizēt _____________________________________
      ii. Nevaru precizēt
   b) Nav izslēgts
   c) Nē

3. Vai pēc bērna apskates jums palicis iespaids / intuitīva sajūta, ka bērnam ir pašlimitējoša saslimšana?
   a) Jā
      Kāpēc:
      1. Varu percizēt _____________________________________
      2. Nevaru precizēt
   b) Neesmu pārliecināts/ta
   c) Nē

4. Balstoties uz anamnēzes un objektīvās izmeklēšanas datiem, lūdzu atzīmēt saslimšanas, kas varētu būt bērnam šajā saslimšanas epizodē:

   a) Ādas un mīksto audu infekcija
   b) Akūta urīnceļu infekcija
   c) Pneimonija
   d) Bakteriāls gastroenterīts
   e) Bakteriāls meningīts
   f) Akūts osteomielīts
   g) Septisks artrīts
   h) Neprecizēta bakteriāla infekcija
   i) Sepse
   j) Nekas no minētā

5. Vai bērna anamnēzē un fizikālās izmeklēšanas datos sastopams kāds no sekojošajiem (atzīmēt esošo)

   a) Toksisks izskats / bērns izskatās smagi slims
   b) Miegainība
   c) Stenēšana
   d) Nepārtraukta raudāšana
   e) Cianoze
   f) Tahipnoe
   g) Elpas trūkums
   h) Mikrocirkulācijas traucējumi
   i) Pozitīvi meningeālie simptomi
   j) Petehiāli izsitumi
   k) Krampji
   l) Arteriāla hipotensija
   m) Bezsamaņa

6. Anketu aizpilda:
   a) Sertificēts ārsts: darba stāžs (gados) ______________________
   b) Ārsts – rezidens: gads _____________
