# Supplementary material for: Integrating Clinical Signs at Presentation and Clinician's Non-analytical Reasoning in Prediction Models for Serious Bacterial Infection in Febrile Children Presenting to Emergency Department
Source: Front Pediatr. 2022 Apr 25;10:786795. doi: 10.3389/fped.2022.786795 (PMC9082163; doi:10.3389/fped.2022.786795)
Supplement: Supplementary file 7 [file Table_1.PDF]

Supplementary Table 1. Reference values for tachycardia and tachypnoea according to age.

Age	Heart rate for tachycardia, beats per minute	Respiratory rate for tachypnoea, breaths per minute
0 to 12 months	> 160	> 60
1 to 3 years	> 150	> 50
3 to 5 years	> 140	> 40
5 to 8 years	> 120	> 30
> 8 years	> 115	> 25
