# Supplementary material for: Integrating Clinical Signs at Presentation and Clinician's Non-analytical Reasoning in Prediction Models for Serious Bacterial Infection in Febrile Children Presenting to Emergency Department
Source: Front Pediatr. 2022 Apr 25;10:786795. doi: 10.3389/fped.2022.786795 (PMC9082163; doi:10.3389/fped.2022.786795)
Supplement: Supplementary file 8 [file Table_2.PDF]

Supplementary Table 2. Interpretation of the clinical score according to outcomes in research and validation populations

Points	Interpretation	Research cohort (CCUH)		Validation cohort Regional hospitals		Total*	
		Non-SBI N** (%)	SBI N (%)	Non-SBI N (%)	SBI N (%)	Non-SBI N (%)	SBI N (%)
0	Low risk	26 (9.8%)	1 (1.3%)	13 (9.7%)	1 (2.0%)	39 (9.8%)	2 (1.6%)
1		61 (23.0%)	4 (5.0%)	31 (23.1%)	1 (2.0%)	92 (23.1%)	5 (3.9%)
2		1 (0.4%)	0 (0.0%)	1 (0.7%)	0 (0.0%)	2 (0.5%)	0 (0.0%)
3		37 (14.0%)	4 (5.0%)	24 (17.9%)	7 (14.3%)	61 (15.3%)	11 (8.5%)
4	"Grey area"	84 (31.7%)	19 (23.8%)	34 (25.4%)	10 (20.4%)	118 (29.6%)	29 (22.5%)
5		33 (12.5%)	19 (23.8%)	8 (6.0%)	8 (16.3%)	41 (10.3%)	27 (20.9%)
6	High risk	8 (3.0%)	12 (15.0%)	14 (10.4%)	6 (12.2%)	22 (5.5%)	18 (14.0%)
7		9 (3.4%)	8 (10.0%)	4 (3.0%)	6 (12.2.%)	13 (3.3%)	14 (10.9%)
8		3 (1.1%)	6 (7.5%)	2 (1.5%)	2 (4.1%)	5 (1.3%)	8 (6.2%)
9		3 (1.1%)	0 (0.0%)	3 (2.2%)	5 (10.2%)	6 (1.5%)	5 (3.9%)
10		0 (0.0%)	7 (8.8%)	0 (0.0%)	2 (4.1%)	0 (0.0%)	9 (7.0%)
11		0 (0.0%)	0 (0.0%)	0 (0.0%)	1 (2.0%)	0 (0.0%)	1 (0.8%)
